# B cell extracellular vesicles influence melanoma response to immune checkpoint therapy

**DOI:** 10.1126/sciadv.adt4551

**Published:** 2025-10-08

**Authors:** Ala’a Al Hrout, Agshin Balayev, Karla Cervantes-Gracia, Konstantinos Gkelis, Stephan Benke, Julia M. Matínez Gómez, Karina Silina, Mitchell P. Levesque, Richard Chahwan

**Affiliations:** ^1^ Institute of Experimental Immunology, University of Zurich, Winterthurerstrasse 190, 8057 Zurich, Switzerland.; ^2^ Institute of Pharmaceutical Sciences, Department of Chemistry and Applied Biosciences, Swiss Federal Institute of Technology (ETH Zurich), 8093 Zurich, Switzerland.; ^3^ Flow Cytometry Facility, University of Zurich, Winterthurerstrasse 190, 8057 Zurich, Switzerland.; ^4^ Department of Dermatology, University Hospital Zurich, University of Zurich, 8091 Zurich, Switzerland.; ^5^ EVIIVE AG, 8057 Zurich, Switzerland.

## Abstract

The immune tumor microenvironment is a dynamic ecosystem where B cells play critical roles in modulating immune checkpoint blockade (ICB) therapy responses. While traditionally seen as passive players in tumor immunity, recent evidence suggests that B cells actively influence antitumor responses. This study examines the role of B cells and their extracellular vesicles (EVs) in melanoma responses to ICB. Retrospective meta-analyses reveal increased B cell enrichment in ICB responders’ pretreatment. Functional assays show that B cell depletion impairs T cell–mediated tumor cytotoxicity. EVs from melanoma tumors were analyzed, identifying miR-99a-5p in CD19^+^ EVs as up-regulated in responders. Silencing miR-99a-5p in B cells reduces T cell antitumor activity, suggesting its role in immune modulation. Mechanistically, miR-99a-5p promotes B cell maturation via class-switch recombination. These findings underscore B cells’ impact on melanoma immunotherapy, offering insights into novel therapeutic strategies targeting B cell–related pathways.

## Teaser

B cells and their extracellular vesicles enhance melanoma immunotherapy by boosting T cell antitumor responses.

## INTRODUCTION

Immune cells, innate and adaptive, represent an essential component of the tumor microenvironment (TME), shaping cancer therapy outcomes. Their dichotomous properties hinge on the balance of tumor-promoting versus tumor-suppressing functions. T cells have long been considered paramount in context of immunotherapy and patient outcome due to their direct antitumor cytotoxicity. T cells represent the most common tumor-infiltrating lymphocytes (TILs) and their enrichment in tumors has been correlated with favorable prognosis (*1*–*3*). However, T cells also depend on B cells for efficient tumor-targeted cytotoxicity. B cells contribute to antitumor immunity through antibody production, cytokine secretion, antigen presentation, and costimulation of auxiliary immune cells (*4*–*6*). Like T cells, tumor-infiltrating B cells could differentiate into several subtypes in response to cues from the TME. The understanding that B cells are a diverse population with a vast range of functions and properties can clarify their dual role in tumor immunity.

In melanoma, B cells can constitute up to half of the TILs population [reviewed in (*7*)]. Studies have shown that they play an essential role in fostering an anti-TME and in activating T cells (*8*). The contributions of B cells in immunotherapy highlight an unexpected role of this overlooked immune cell population in shaping the TME. Several studies reported a strong correlation between B cells enrichment and localization with immune checkpoint blockade (ICB) therapy outcome in melanoma (*9*–*12*). The presence of B cells in melanoma biopsies before ICB treatment correlated with positive outcomes (*10*, *11*). In addition, their colocalization with CD8^+^ T cells was linked to improved overall survival (OS), independent of other clinical variables (*10*). Tumor-associated B cells can localize within tertiary lymphoid structures that highly resemble secondary lymphoid organs and lymph nodes in their formation and organization (*7*). Nonetheless, tumor-associated B cells can also localize within the stroma or tumor margins, hinting that such B cell–mediated effects could be achieved, in part, by proxy signaling. This could be mediated by classical B cell signaling such as antibody secretion and cytokines, but an additional enticing possibility is the involvement of extracellular vesicles (EVs).

EVs are emerging as complex signaling mediators within the TME. Tumor-derived EVs have been implicated in several stages of the tumorigenesis process, by regulating proliferation, angiogenesis, and immune-suppression (*13*). EV-mediated resistance has been described in melanoma through shuttling of tumor-derived PDL-1 on EVs (*14*–*17*), suggesting a substantial contribution of EVs in shaping immunotherapy outcome. Conversely, immune cells can regulate each other via EV-mediated signaling to promote immune-activation or immune-suppression based on TME cues. EV cargo contains proteins and small noncoding RNA including microRNA (miRNAs). Like gene expression, miRNAs can be differentially expressed (DE) between cells; hence, EV-derived miRNAs also reflect that difference among different cell populations, even between EVs and their parent cells (*18*). This suggests that EV cargo packaging and their transport is not a passive process, but an active and controlled one. Hence, we postulate that EVs can carry signature miRNAs that can elucidate the contribution of certain cell populations in the context under investigation, for example, ICB therapy response. Despite the exciting studies reporting B cells role in promoting immunotherapy, most studies done are retrospective, relying on outcome post therapy. To gain more understanding, it is necessary to move from clinical data to mechanisms, which can ultimately translate into prognostic and therapeutic benefits. In this study, we explore the role of B cell–derived EVs in shaping the TME of ICB responders versus nonresponders. We studied the in situ EVs profile from melanoma patients undergoing ICB therapy—from a cohort collected at baseline—through isolating tissue-derived EVs. EVs faithfully recapitulate the TME, where ICB therapy responders and nonresponders can be divided into two cohorts based on their EVs surface markers and cargo, as if EVs had a “fingerprint” that convey their origin and microenvironmental context. Nonetheless, bulk EVs sequencing can be misleading, or at best, uninformative when looking at subtle changes in specific subpopulations within the whole EV population. Therefore, melanoma and B cell–derived EVs were individually isolated from the heterogenous mixture of bulk EVs, and their miRNA cargo was isolated and sequenced. Strikingly, the EVs landscape reflected what we and others have observed at the cellular level, of enrichment of immune cell infiltration in responders over nonresponders. B cell–derived EVs had far more unique miRNAs that are not shared with melanoma EVs, highlighting that B cells exhibit notable change in the context of ICB therapy. miR-99a-5p, a well-established tumor suppressor, was identified in B cell–derived EVs from responders. In downstream functional assays we were able to show that miR-99a-5p exhibits a B cell–dependent phenotype, where it regulates the cell cycle and DNA damage repair pathways, resulting in enhanced class-switch recombination (CSR). We propose that B cells in ICB therapy responders exhibit higher CSR and enhanced antitumor response via EV-based signaling, in part, via miR-99a-5p. These findings collectively suggest that B cells and their derived EVs are remarkable contributors to shaping therapeutic outcome in context of ICB therapy in melanoma.

## RESULTS

### B cells are enriched in ICB therapy responders pre-treatment

**Fig. 1.**
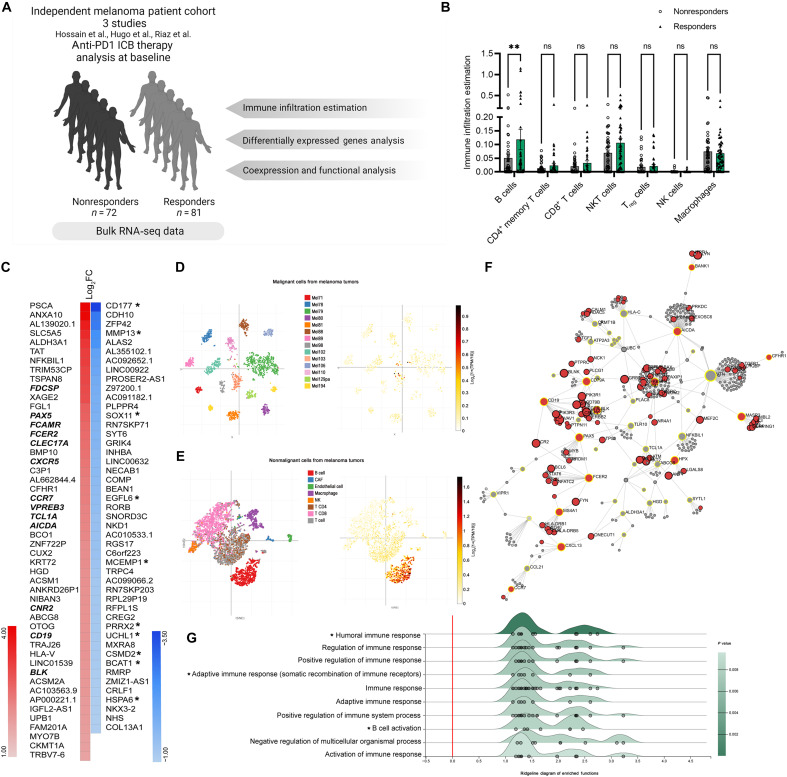
B cells are enriched in the tumors of ICB therapy responders in cohorts of patients with melanoma. (**A**) Schematic depiction of datasets design for meta-analysis. (**B**) Immune infiltration estimation based on bulk RNA-seq data. Estimation was done using TIMER2.0. *P* values were determined by two-way analysis of variance (ANOVA) with Sidak multiple comparison test (*P* < 0.004). (**C**) Expression heatmap of the top 50 significant DEGs in responders in comparison to nonresponders. Asterisks signify down-regulated genes in responders involved in tumor progression and metastasis. (**D**) tSNE plot of scRNA-seq of malignant cells from melanoma tumors and expression location of the top 100 significant up-regulated genes (*22*) visualized on Single Cell portal (Broad Institute). (**E**) t-distributed Stochastic Neighbor Embedding (tSNE) plot of scRNA-seq of nonmalignant cells from melanoma tumors and expression location of the top 100 significant up-regulated genes visualized on Single Cell portal (Broad Institute). (**F**) Protein-protein interaction network of significant up-regulated genes in responders over nonresponders (encircled in yellow) and their immediate network. Proteins in red are involved in BCR signaling pathway (full list in table S1). Network is visualized using NetworkAnalyst (*74*). (**G**) Ridgeline diagram of enriched biological process of the significant up-regulated genes. *x* axis shows expression fold change of responders over nonresponders. Ridgeline analysis was done with ExpressAnalyst (*72*).

Our meta-analysis of large-scale RNA sequencing (RNA-seq) data from three independent melanoma studies (*19*–*21*) (total *n* = 153) at baseline before undergoing anti-PD1 ICB therapy, provided insights into the role of B cells in shaping the melanoma response to immunotherapy by comparing responder (*n* = 81) versus nonresponder (*n* = 72) cohorts (Fig. 1A). The estimation of immune infiltration in ICB responders over nonresponders revealed a significant enrichment of B cells (Fig. 1B). This unexpected finding was further corroborated by analyzing differentially expressed genes (DEGs) between responders and nonresponders. The top 50 significant DEGs are shown in Fig. 1C. Significantly up-regulated DEGs in ICB therapy responders predominantly included genes specifically expressed by B cells and/or influencing B cells development and homing, including *CD19*, *AICDA*, *PAX5*, and *BLK* (Fig. 1C, bold). On the other hand, the top down-regulated genes in responders included genes involved in tumor progression and metastasis in melanoma and other cancers (Fig. 1C, asterisk). Validation in a fourth independent dataset of single cell RNA-seq (scRNA-seq) data of melanoma tumors (*22*) visualized on Single Cell portal (Broad Institute) corroborated these findings; expression of significant up-regulated DEGs from our meta-analysis was enriched in the B cells cluster rather than the melanoma cluster (Fig. 1, D and E). Furthermore, protein-protein network analysis of significant up-regulated genes (encircled in yellow) and their immediate protein neighbors highlighted proteins that shape B cell responses as a major contributor to the network (Fig. 1F). These proteins (in red) play a role in B cell activation, differentiation, somatic recombination of immune receptors [immunoglobulin (Ig) superfamily], and B cell receptor (BCR) signaling (tables S1 and S2). To understand the function of the up-regulated genes in responders, we carried out gene ontology analysis of biological process term enrichment. As predicted, and in concordance with our earlier results, the enriched terms generally included immune response regulation, in addition to somatic recombination of immune receptors and B cell activation (Fig. 1G).

### T cell antitumor cytotoxic activity is impaired by B cell depletion

**Fig. 2.**
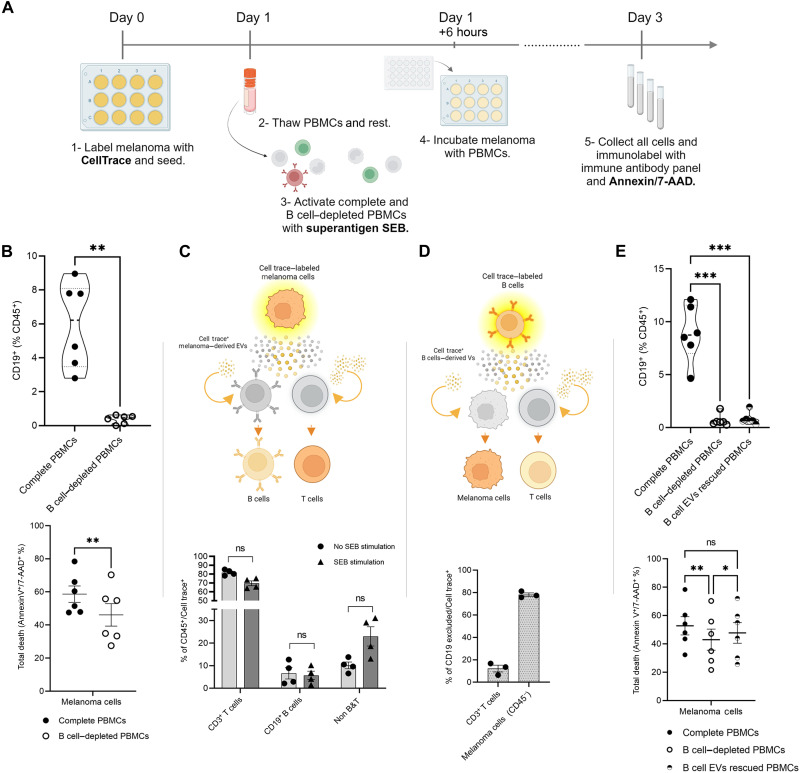
Cell-cell signaling is mediated through EVs signaling. (**A**) Schematic depiction of tumor-killing assay setup. (**B**) CD19^+^ cells in complete and B cell–depleted cultures. Presented as percentage of CD45^+^ population. *P* values were determined by two-tailed paired *t* test (**P < 0.005). Tumor-killing assay in complete and B cell–depleted cultures, presented as total death of melanoma cells that are single and double positive to annexin-V–FITC and 7-AAD. *P* values were determined by paired *t* test (**P < 0.005). (**C**) EVs uptake from CT–stained melanoma cells. CD3^+^, CD19^+^, and CD3^−^CD19^−^ populations out of CD45^+^/CT^+^ with and without SEB stimulation, presented as percentile. *P* values were determined by Sidak multiple comparisons *t* test. (**D**) EV uptake from CT–stained B cells. Melanoma population and CD3^+^ population out of CD19 excluded/CT^+^ without SEB stimulation, presented as percentile. (**E**) CD19^+^ cells in complete, B cell–depleted, B cell EV–rescued cultures. Presented as percentage of CD45^+^ population. *P* values were determined by one-way ANOVA (***P < 0.001). Tumor-killing assay in complete, B cell–depleted, B cell EV–rescued cultures, presented as total death of melanoma cells that are single and double positive to annexin-V–FITC and 7-AAD. *P* values were determined by one-way ANOVA (*P < 0.05, **P < 0.01).

To address our initial observation that B cells might play an important role in T cell–mediated tumor killing in melanoma, we developed a coculture tumor-killing assay. In our experimental setup (Fig. 2A), we first label melanoma cells with CellTrace (CT) to distinguish them from the remainder of the peripheral blood mononuclear cell (PBMC) coculture. Activation of PBMCs (from healthy donors) with staphylococcal enterotoxin B (SEB) cross-links T cell receptor (TCR) with major histocompatibility complex (MHC) II on antigen presenting cells and bypasses the need for specific TCR-MHC–peptide complex interaction to elicit a strong immune response (*23*–*25*). We then compare cell death of melanoma cells (positive for annexin V/7AAD) when cocultured with complete or B cell–depleted PBMCs (Fig. 2B, top). B cell depletion resulted in a significant decrease in melanoma’s cell death in comparison to complete PBMCs culture (Fig. 2B, bottom) without affecting CD3^+^ T cell numbers (fig. S1A). This suggests that T cell antitumor cytotoxic activity might be impaired by B cell depletion in melanoma samples.

### TME cell-cell signaling can be mediated by EVs

In our tumor-killing assay setting, we observed a CD45^+^ population that is, unexpectedly, also positive for CT used to exclusively stain melanoma cells (fig. S1B). Since CT is quickly inactivated by cell culture media, it could not have escaped from stained melanoma cells to immune cells. Yet, some of the expected CD45^+^/CT^−^ population have uptaken CT stain from melanoma and became CD45^+^/CT^+^. Much of this double-positive population were CD3^+^ T cells, but CD19^+^ B cells were also stained (Fig. 2C and fig. S1C). Similarly, we isolated B cells from PBMCs and tagged them with CT to be cocultured with the rest of PBMCs and melanoma cells (Fig. 2D and fig. S1D). Most non–B cells positive for CT were melanoma cells, suggesting an active and deliberate, rather than passive, CT uptake (Fig. 2D). This is in line with the ability of EVs to transfer cargo across cells especially in the TME (*26*), while there is a possibility that, at least in part, CT could have been transferred between cells because of trogocytosis. To address this, we validated that secreted EVs from CT-tagged cells are themselves CT^+^ (fig. S1E). To further confirm the possible role of B cell–derived EVs in T cell–mediated antitumor activity in the tumor-killing assay, we depleted PBMCs of B cells and then rescued the culture with B cell–derived EVs (Fig. 2E). Rescuing PBMCs with B cell–derived EVs was sufficient to reinstate tumor-killing ability even in the absence of B cells (Fig. 2E). We were intrigued to pursue the interesting observation we made in fig. S1A, where the number of T cells was not affected by B cell depletion, but, nonetheless, it translated into impaired cytotoxicity against melanoma cells (Fig. 2B). We postulated that this is due to reduced T cell activation and, therefore, reduced cytotoxic activity. We assessed the expression of CD69, an early lymphocyte activation marker, on the surface of T cells. Expression was assessed in CD8^+^ cytotoxic T cells cultured with complete PBMCs, B cell–depleted PBMCs, and B cell–EV–rescued PBMCs. As postulated, B cell depletion resulted in significant reduction in CD69 expression in CD8^+^ T cells, whereas B cell–derived EVs were able to rescue CD69 expression in CD8^+^ T cells (fig. S1F).

### In situ EVs highlight differences between ICB responders versus nonresponders

**Fig. 3.**
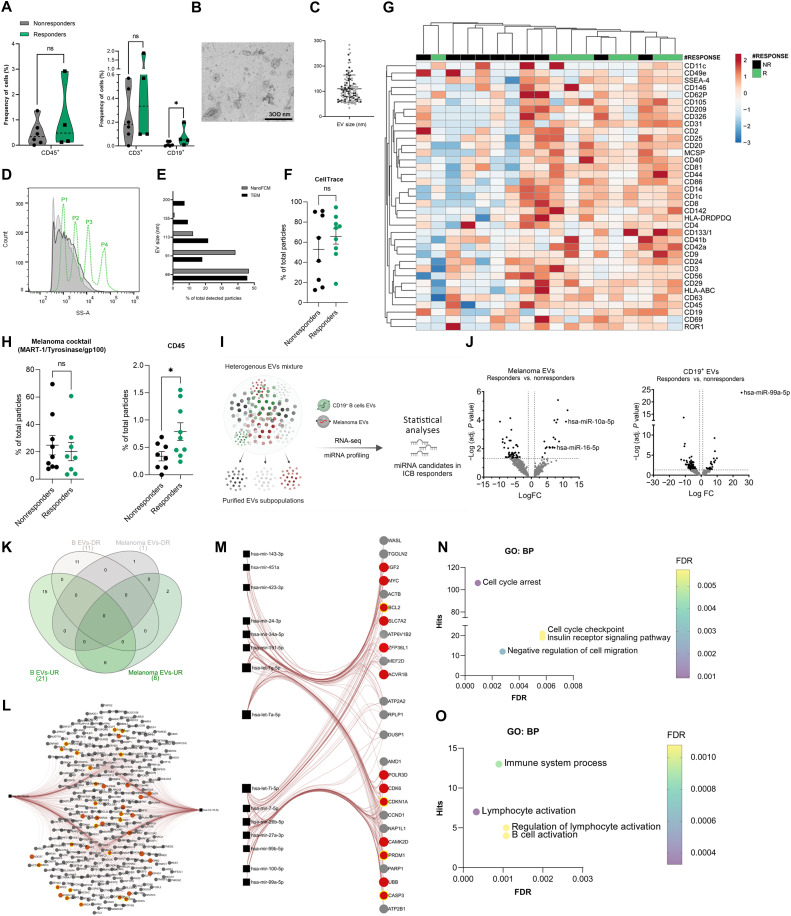
In situ EVs analysis highlights differences between ICB responders and nonresponders. (**A**) CD45^+^, CD3^+^, CD19^+^ and populations in responders and nonresponders processed tissue, presented as frequency of total cells. *P* values were determined by two-sided Mann-Whitney test *U* test (*P < 0.05) ns, not significant. (**B**) TEM representative image of isolated EVs from patient tissue biopsies (**C**) size distribution of particle visualized by TEM images. Analysis was done using MAPS software. (**D**) Size distribution of isolated EVs (light and dark gray, *n* = 2) in comparison to silica sizing nanospheres (dotted green line). Samples were acquired using NanoFCM. (**E**) Comparison of TEM and NanoFCM EVs sizes. (**F**) CT^+^ EVs, presented as percentage of particles in responders (green, *n* = 9) and nonresponders (black, *n* = 8). Each point is an independent patient sample. (**G**) Expression heatmap of denoted surface markers on EVs from responders (*n* = 8) and nonresponders (*n* = 10). Original values are ln (x + 1) transformed. Rows are clustered using Euclidean distance and average linkage. Columns are clustered using maximum distance and average linkage. Created with ClustVis (*76*). (**H**) Melanoma cocktail^+^ (MART-1, tyrosinase, and gp100) and CD45^+^ EVs, presented as percentage of particles in responders (green, *n* = 9) and nonresponders (black, *n* = 6). *P* values were determined by Welch’s *t* test (*P < 0.05). (**I**) schematic depiction of EVs fractionation setup. Created in BioRender. Al Hrout, A. (2025) https://BioRender.com/ve9az06. (**J**) Volcano plots of DE miRNAs in responders (*n* = 8) over nonresponders (*n* = 6) from melanoma and CD19^+^ EVs. (**K**) Venn diagram of significant up-regulated (UR) and down-regulated (DR) miRNAs in responders over nonresponders from melanoma and CD19^+^ EVs. Created with InteractiVenn (*77*). (**L** and **M**) Melanoma-derived EVs (L) and CD19^+^ EVs (M) unique up-regulated miRNAs in responders and their predicted targets (using miRnet 2.0) (*71*). (**N** to **O**) Gene enrichment analysis of selected biological processes associated with predicted gene targets of melanoma-derived EVs (N) and CD19^+^ EVs (O) unique up-regulated miRNAs in responders.

To better replicate in vivo and in situ EV–based signaling within the TME, we explored the isolation of EVs from patient-derived tumor tissue; we had previously shown that melanoma biopsies have abundant tumor and immune EV particles (*27*). With this added approach, we were able to further fractionate and analyze EVs derived from the different TME cell populations resident at the tumor site at the time of biopsy sampling. Using flow cytometry to quantify cells directly from our melanoma biopsy cohort (*n* = 10), we observed a significant enrichment of B cells in melanoma biopsies from anti-PD1 ICB therapy responders versus nonresponders (Fig. 3A). This result supported our meta-analysis of the three independent melanoma datasets. Tissue-derived EVs were then isolated, processed, and imaged using transmission electron microscopy (TEM) to verify their size and morphology (Fig. 3, B and C), as elaborated in our prior work (*27*). The isolated particles displayed expected EV characteristics including: (i) size, ranging between 50 and 180 nm and (ii) morphology, a round concaved bilayer morphology (Fig. 3, B and C). After we established the successful isolation of EVs, we carried out size estimation of the particles using nano-flow cytometry (NanoFCM) (*27*–*29*). NanoFCM sized the isolated EVs accurately, binning them into subpopulations of different sizes, reflecting the size range observed by TEM (Fig. 3, D and E). The quality of our EV prep was further verified using NanoFCM and quality controls to exclude background noise and nonspecific staining (fig. S2A). Our EV isolation displayed high purity, averaging between 60 and 90% as measured by CT, excluding non-biological debris or nonmembranous particles (i.e., exomeres or supermeres) (Fig. 3F). Staining for CD63 also showed a high percentage, further confirming EV isolation efficacy (fig. S2B).

Multiplex EV surface protein detection gave us a comprehensive overview of tissue-derived EVs in responders and nonresponders, including immune cell–derived EVs. Overall, tissue-derived EVs from responders had a higher expression of immune markers (Fig. 3G). Hierarchical-clustering analysis of the heatmap showed a segregation between responders and nonresponders, highlighting that tissue-derived EVs recapitulated what we have observed thus far in the cellular TME of immune populations enrichment in responders over nonresponders, underscoring the reliability of tissue-derived EVs in representing the in situ TME. This observation was further corroborated with the analysis performed using NanoFCM detection of melanoma and immune-derived EVs on the single-particle level. Immune cell–derived EVs (CD45^+^) were significantly higher in responders over nonresponders (Fig. 3H), whereas melanoma EVs did not show a significant difference between the two cohorts, suggesting that a substantial change occurs on immune cell–derived EVs rather than melanoma-EVs in the context of anti-PD1 ICB therapy.

### B cell–derived EVs influence melanoma response to ICB therapy

Using the results we have achieved thus far, we aimed at untangling the complex network of EVs by fractioning the heterogeneous mixture of tissue-derived EVs into subpopulations based on their surface markers (Fig. 3I). The miRNA cargo of the subpopulations of interest (i.e., B cell– and melanoma-derived EVs) was profiled. We compared each EV subpopulation between responders and nonresponders (e.g., CD19^+^ responders versus CD19^+^ nonresponders). On the basis of differential expression analysis, miRNAs that met the cutoff threshold (Log_2_FC ≥ 1, FDR < 0.05) were defined as significant DE miRNAs (Fig. 3J). When comparing ICB responders versus nonresponders, 21 miRNAs were up-regulated in CD19^+^ EVs, and 8 miRNAs in melanoma EVs. Excluding the possibility of technical biases toward sequencing readouts, this might suggest that changes in miRNA cargo in response to ICB therapy are more pronounced in B cell–derived EVs than in melanoma cell–derived EVs. This is especially intriguing as CD19^+^ EV percentages are not significantly different between responders and nonresponders (fig. S2C). Therefore, we postulate that DE miRNAs that are unique to B cell–derived EVs could shed more light on the mechanistic role of B cells in shaping the response to ICB. As a result, DE miRNAs in responders versus nonresponders of B cell–derived and melanoma-derived EVs were plotted in a Venn diagram (Fig. 3K).

We explored the predicted gene targets of the unique miRNAs identified to be up-regulated in melanoma-derived EVs (Fig. 3L) and B cell-derived EVs in responders (Fig. 3M). Melanoma-derived EVs had only two unique miRNAs that are not shared with B cell–derived EVs. Predicted gene targets of these unique miRNAs were enriched in cell cycle arrest and cell cycle checkpoint pathways (Fig. 3N). The predicted targets of B cell–derived EVs miRNAs were enriched in immune system process pathway, lymphocyte activation pathway, and B cell activation pathways (Fig. 3O). Given the scope of this study, we decided to focus on B cell–derived EVs miRNAs for our mechanistic studies.

### miR-99a-5p of B cell EVs promote melanoma ICB response

miR-99a-5p was the top hit among the 15 unique miRNAs identified in B cell–derived EVs in responders. miR-99a has been established as a master tumor suppressor across many cancers (*30*–*33*). However, the expressing cell and the mechanism by which miR-99a-5p exerts its tumor-suppressing function remains unknown. Herein, we propose that miR-99a-5p could play a critical role in the antitumor response B cells play in context of melanoma ICB therapy.

**Fig. 4.**
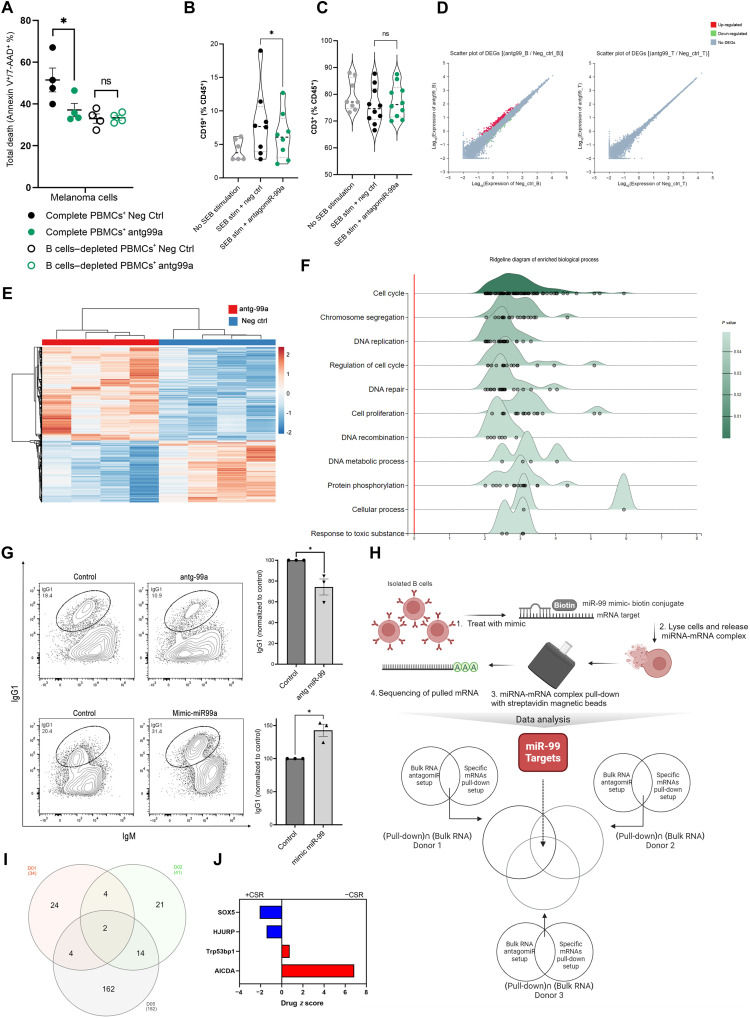
miR-99a-5p B cell–mediated phenotype is through regulation of the cell cycle and CSR. (**A**) Tumor-killing assay in complete and B cell–depleted cultures treated with antagomiR-99a-5p or negative control antagomiR, presented as total death of melanoma cells that are single and double positive to annexin-V–FITC and 7-AAD. *P* values were determined by two-tailed paired *t* test (*P < 0.05). (**B** and **C**) CD19^+^ B cells (B) and CD3^+^ T cells (C) with or without SEB stimulation and treated with antagomiR-99a-5p or negative control antagomiR. Presented as percentage of CD45^+^ population. *P* value was determined by two-tailed paired *t* test (*P < 0.05). (**D**) Scatter plot of DEGs in isolated B cells and T cells treated with antagomiR-99a-5p or negative control antagomiR (*n* = 4). (**E**) Hierarchical heatmap clustering of transcriptome profiles of isolated B cells treated with antagomiR-99a-5p or negative control antagomiR (*n* = 4). Rows are centered; unit variance scaling is applied to rows. Columns are clustered using correlation distance and average linkage. Created with ClustVis (*76*). (**F**) Ridgeline diagram of enriched biological process of significant up-regulated genes. *x* axis shows expression fold change of antagomir-99a-rp–treated cells over negative control antagomiR. Ridgeline analysis was done with ExpressAnalyst (*72*). (**G**) IgG1^+^ B cells in antagomiR-99a-5p treated normalized to negative control antagomir. *P* values were determined by two-tailed paired *t* test. (**H**) Schematic depiction of experimental setup for miRNA pull-down and downstream analysis plan for identification of miR-99a-5p direct targets based on pull-down RNA-seq and bulk RNA-seq. (**I**) Venn diagram of up-regulated genes pull-down seq ∩ up-regulated genes bulk RNA-seq from all three donors (where ∩ denotes intersection of datasets). (**J**) *z*-score bar graph of Genome-wide CRISPR knockout screen data in CH12F3-2 cells from Feng *et al.* (*35*). Positive *z* score indicates reduced CSR, and negative *z* score indicates enhanced CSR upon the knocking-out of denoted genes.

To that end, we treated PBMCs with miR-99a-5p silencer (antagomir-99a) or with negative control. We compared complete PBMCs and B cell–depleted PBMCs killing ability with silencing miR-99a-5p (Fig. 4A). Silencing miR-99a-5p significantly impaired tumor-killing ability, and only in the presence of B cells where the strong phenotype was lost upon B cell depletion (Fig. 4A). Silencing miR-99a-5p significantly reduced the percentage of CD19^+^ B cells but did not affect the percentage of CD3^+^ T cells (Fig. 4, B and C).

To understand the mechanism by which miR-99a-5p exerts its function and to confirm that it is B cell dependent, we silenced miR-99a-5p in isolated B and T cells. Treatment of B cells, but not T cells, with antagomiR-99 resulted in significant differential gene expression (Fig. 4D), thereby corroborating our previous findings of a B cell–dependent phenotype upon silencing miR-99a-5p. Hierarchical clustering heatmap highlighted clusters of genes that are associated with miR-99a-5p silencing (Fig. 4E). DEG analysis (Log_2_FC ≥ 1 and *q* value < 0.05) revealed 155 significantly up-regulated genes and 15 down-regulated genes upon antagomir-99 treatment versus negative control. The 20 top up-regulated genes and all down-regulated genes are shown in fig. S3A (full list in table S3). Enrichment analysis of significantly up-regulated genes highlighted the cell cycle as one of the top pathways affected (Fig. 4F). Up-regulated genes were also enriched in DNA repair signaling. Both of which are pathways involved in B cell class switch recombination (CSR), a process by which diversification of antibody isotypes is mediated. Biological process enrichment analysis (fig. S3B), supported by up-regulation of key cell cycle regulators (fig. S3C) showed a similar trend, thereby supporting cell cycle regulation as a main arm of miR-99a-5p function. miR-99a-5p–mediated gene regulation also seems to favor nonhomologous end joining (NHEJ) protein machinery over homologous recombination (HR) machinery, the former being primarily used by CSR. This is exhibited through the up-regulation of HR key players including *EME1*, *RAD51*, and *DMC1*, among others, upon silencing of miR-99a-5p (fig. S3D). In addition, *PLXND1*, which is described as a novel regulator of germinal centers and humoral immune responses (*34*), was down-regulated upon miR-99a-5p silencing in our setting. Next, we wanted to explore whether miR-99a-5p indeed regulates CSR efficiency based on the aforementioned mechanisms. We treated splenic B cells with a silencer or a mimicker of miR-99a-5p with their respective controls and assessed class switching from IgM to IgG1. Strikingly, silencing miR-99a-5p resulted in a significant decrease in CSR efficiency, exhibited by lower IgG1 detection, while overexpressing miR-99a-5p with a miRNA mimic resulted in a significant increase in CSR (Fig. 4G).

### miR-99a-5p regulates B cell maturation by targeting CSR suppressors

While silencing and mimicking miR-99a-5p gave us an idea of the possible genes and pathways regulated by B cell EV-miRNA signaling, it did not provide a specific downstream target mediating such function. We therefore aimed to identify miR-99a-5p direct binding targets to understand the mechanism by which it mediates its B cell phenotype using a streptavidin-bead–based pull-down assay. After treating isolated B cells with a biotinylated mimic of miR-99a-5p and biotinylated negative control, we lysed the cells and collected the miRNA-mRNA complexes with streptavidin-conjugated beads. By comparing up-regulated genes in mimic-miRNA versus negative control-miRNA pulldown, we were able to identify possible targets of miR-99a-5p. Up-regulated genes in the pull-down prep were then compared to up-regulated genes in bulk RNA-seq prep (± miR-99a-5p silencing) in each respective donor (Fig. 4H). The rationale is that up-regulated genes upon silencing miR-99a-5p should be targets of miR-99a-5p (directly or indirectly), and by comparing them to pull-down genes, we would be able to eliminate the hits that are not directly targeted by miR-99a-5p. The overlap between all comparisons resulted in two unique hits, namely *HJURP* and *SOX5* (Fig. 4I). The role of these two genes in CSR has been independently verified in a genome-wide CRISPR knockout screen study by Feng *et al.* (*35*). *HJURP* and *SOX5* knockout lines showed more class switching than wild-type lines suggesting that they are suppressor of CSR. Drug *z* scores of *AICDA* and *TP53BP1*, well-established regulating genes in CSR, in comparison to *HJURP* and *SOX5* are shown in Fig. 4J. *z* scores are obtained from (*35*), where positive *z* score indicates reduced CSR and negative *z* score indicates enhanced CSR upon the knocking-out of denoted genes. HJURP and SOX5 are likely suppressing CSR through distinct but complementary mechanisms. *HJURP’s* role in chromatin remodeling prevents the necessary DNA regions from becoming accessible for CSR, while *SOX5*’s role in transcriptional regulation ensures that the regulation and fine-tuning expression of genes required for CSR. Together, they create a robust system to maintain genomic stability, thereby preventing the necessary DNA recombination required for CSR.

### B cell isotype switching positively correlates with better prognosis

**Fig. 5.**
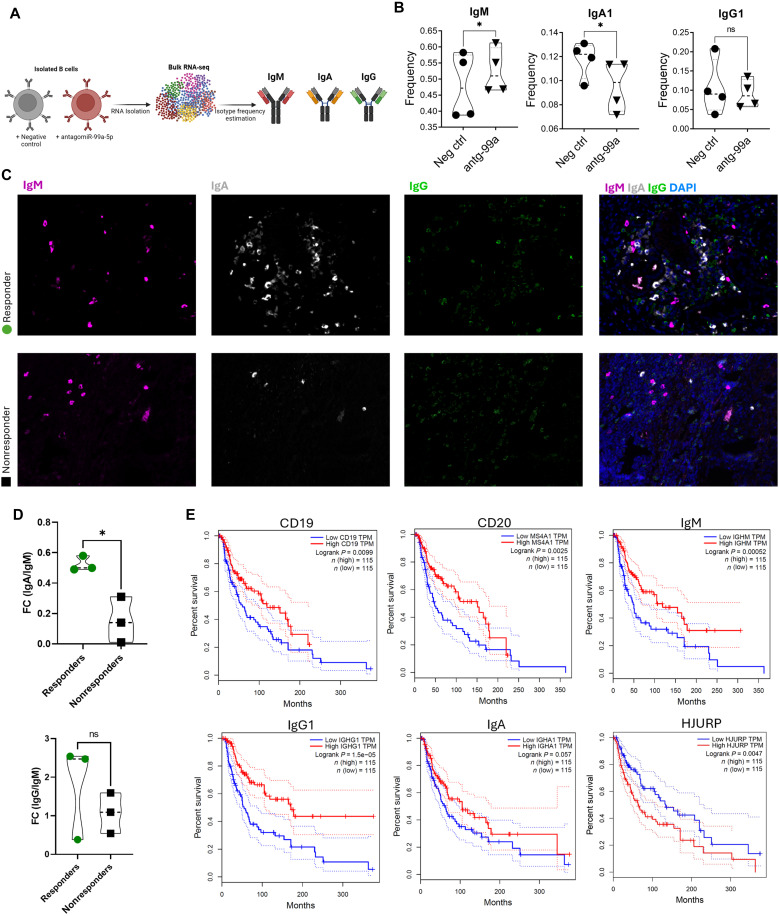
Switched Igs are associated with increased OS. (**A**) Graphical description of data shown in (B). (**B**) Quantification of Ig isotype frequency in PBMC-derived B cells bulk RNA-seq data (Fig. 4, E to F). Cells were treated with negative control antagomir or antagomir against miR-99a-5p. (**C**) Representative images of immunofluorescence staining for IgM (magenta), IgG (green), and IgA (gray) from responders and nonresponders. (**D**) Quantification of Ig switching, represented as fold increase of IgA or IgG over IgM. *P* values were determined by Welch’s *t* test (*P < 0.05). (**E**) OS plots of melanoma patients (SKCM dataset) using GEPIA platform (*75*) (Mantel-Cox test for hypothesis test). Survival is plotted as a function of *CD19*, *CD20*, *IgM*, *IgG1*, *IgA*, and *HJURP* expression.

By analyzing Ig isotype frequency in our PBMC-derived B cells, we were able to show that miR-99a-5p regulates CSR (Fig. 5, A and B). Silencing miR-99a-5p results in a significant up-regulation of IgM, and a significant down-regulation of IgA (Fig. 5B). This further confirms that the conclusion we drew from our transcriptome analysis of DEGs in human B cells treated with antagomir-99 (Fig. 4, D to F) was indeed valid. Therefore, and on the basis of our earlier data, we expected that we should observe increased Ig isotype switching in ICB therapy responders over nonresponders, as they express significantly higher miR-99a-5p in their B cell-EVs. This was the case in biopsy samples from patients with melanoma, where responders showed a significant increase in isotype class-switching to IgA, and an increase in switching to IgG, while statistically insignificant (presented as IgG or IgA fold increase over IgM) in comparison to nonresponders (Fig. 5, A to D). We postulate that increased class-switching enhances the antitumor immune response and could play a role in immune-surveillance. BCR diversification reflects a highly responsive and adaptable immune system. A diversified BCR repertoire allows for broader and more effective antigen recognition, improved antibody production, better T cell activation, and sustained immune pressure on the tumor, all of which contribute to the favorable immunotherapy response and better clinical outcomes.

This was in line with independent TCGA data, where correlation analysis was done to analyze the relationship between CD19, CD20, and Ig expression and the prognosis of melanoma by using Kaplan-Meier survival curve analysis (Fig. 5E). The results showed a significant positive correlation between the high expression of these genes and good prognosis. Whereas analysis of HJURP showed a significant positive correlation of low expression of HJURP with better survival (Fig. 5E). In addition, we were able to observe a trend of increased enrichment of CD19^+^CD20^+^ cells in tissues of responders when compared to nonresponders (fig. S4). These findings show that (i) increased presence of B cells (marked by high expression of CD19 and CD20) and enhanced antibody production (marked by high-expression Igs) is correlated with better prognosis in melanoma, and (ii) lower expression of HJURP, a direct target of miR99, is correlated with enhanced survival. Collectively, these findings suggest a direct role of B cell EVs in modulating the immune TME through enhancing CSR and promoting a proimmune, tumor-hostile TME.

## DISCUSSION

We propose that the response to therapy relies on a specific immune cell profile at baseline, in addition to tumor-derived antigens and broad activation of T cells through ICB therapy. This conclusion can be drawn from studies showing that (i) B cell–associated signatures were enriched in immunotherapy responders over nonresponders, (ii) B cell signatures were more prominent than T cell signatures, and (iii) B cell infiltration and BCR diversification are independent OS prognostic factors in melanoma (*36*–*39*). These observations were corroborated by our meta-analysis of three independent datasets exploring transcriptome profile differences between responders and nonresponders of anti-PD1 therapy at baseline (*19*–*21*). We were able to show that there is a correlation between B cell tumor enrichment and response outcome. Most of the up-regulated genes in responders include many genes influencing B cell development, homing, and antibody diversification such as *AID*, *PAX5*, and *CXCR5/CXCL13* (*36*–*39*), and where T cell signatures were far less present. Similar findings of B cells enrichment in responders’ tumor tissue were reported by Helmink *et al.* (*11*). In a fourth independent scRNA-seq dataset, we were able to show that the up-regulated genes in responders (from the meta-analysis) are enriched in the B cell cluster rather than in melanoma cells or any other immune compartment. In addition, we show that these up-regulated genes have distinct B cell–based functions, including B cell activation, differentiation, somatic recombination of immune receptors (Ig superfamily), and BCR signaling. Collectively these data strengthen the correlation between B cells and ICB therapy outcome and gives support to the notion that substantial change in the TME of responders happens at the B cells level.

Optimal T cell antitumor immune response requires B cell presence and contribution. Reports have clearly shown depletion of B cells to decrease CD4^+^ and CD8^+^ T cells infiltration, impair their activation, and increase tumor burden (*8*, *9*, *40*). However, some studies have reported variations in B cell counts and their association with PD-1 inhibition outcomes, indicating complexity in this relationship (*41*). Despite some variability in reported findings, our data support the role of B cell depletion in modulating T cell responses. In our in vitro tumor-killing assay, we were able to reproduce B cell depletion effect on impairing T cell–mediated antitumor activity. Depletion of B cells did not affect the total percentage of T cells in culture, so we postulated that the impairment in T cell–mediated cytotoxicity upon B cell depletion was due to noncellular B cell–based mechanism. In addition, we observed a transfer of fluorescent stain from labeled cells to nonlabeled cells in our coculture system. We were able to show that this transfer could be via the uptake of EVs from labeled cells by recipient cells. We also showed that this transfer is not passive, but deliberate, as there’s a clear differential uptake of fluorescent stain by different cell types in the coculture system. We were also able to show that rescuing B cell–depleted cultures with B cell–derived EVs was able to rescue T cell–mediated antitumor activity through restoring CD8^+^ T cells activity. Collectively, this strongly supports our premise, that B cells exercise their antitumor role, at least in part, via EV-mediated signaling. This led us to move toward studying in situ EV-mediated signaling in melanoma patient tumors undergoing ICB therapy. Here, we show the direct role of tumor in situ CD19^+^ EVs in shaping response to ICB therapy. Our analysis of tumor biopsies of patients with melanoma reproduced the same conclusion as previous reports showing an enrichment of B cells within the tumors of responders (*11*). Strikingly, tissue-derived EVs recapitulated what we have observed thus far in the cellular TME of immune populations enrichment in responders over nonresponders. This also resulted in distinct clustering of EV profiles from patients into two groups, which correlated with the immunotherapy response of the patients. Notably, this clustering was independent of the presence of tertiary lymphoid structures in patients’ tumors.

By comparing the miRNA cargo of B cell–derived EVs with melanoma-derived EVs, we were able to identify a set of miRNAs unique to B cell–derived EVs. The predicted gene targets of the unique B cell–derived EVs miRNA, up-regulated in responders, seem to play a role in lymphocytes activation, suggesting a possible mechanism of self-regulation and activation. The top up-regulated miRNA in responders CD19^+^ EVs was miR-99a-5p, a well-established tumor suppressor across multiple cancers (*31*, *32*, *42*, *43*). We propose that miR-99a-5p is selectively packaged in CD19^+^ EVs in responders, and it plays an important role in B cell–mediated anti–tumor role. Therefore, this led us to postulate that silencing miR-99a-5p could lead to impaired tumor killing in our coculture system. Treatment with antagomiR-99a-5p was able to significantly reduce tumor killing in our coculture system. Moreover, antagomiR-99a-5p impaired T cell–mediated antitumor activity only in the presence of B cells, and without affecting the number of T cells in culture, further confirming miR-99a-5p B cell–dependent role.

We aimed to identify possible targets of miR-99a-5p regulation. Isolated B and T cells were treated with anitagomiR-99a-5p to silence it and sequenced for transcriptome profiling. B cells had several significant DEGs, whereas T cells had no significant DEGs, reiterating that miR-99a-5p has a B cell–dependent phenotype. On the basis of transcriptome data, miR-99a-5p regulates the cell cycle progression by inhibiting key cell cycle regulators, either directly or indirectly. miR-99a-5p also regulates genes involved in DNA repair and recombination, both of which are parts of the CSR process. We propose that miR-99a-5p regulates the cell cycle through prolonging G_1_ phase to promote the NHEJ repair pathway and subsequently promote CSR. This notion could be partially supported by the up-regulation of E2F family genes, *CDC25A*, *CDK1*, and cyclin E2 (*CCNE2*), among other factors known to regulate G_1_-S phase transition, upon miR-99a-5p silencing (*44*–*47*). This has been independently verified in RCC cells, where expressing miR-99a-5p exogenously induced G_1_-phase cell cycle arrest through targeting several cyclins in RCC cells (*48*). CSR is shown to be more active in G_1_ to early S phase as DNA is more accessible and DNA repair factors in CSR are cell cycle–dependent (*49*, *50*) miR-99a-5p might also favor NHEJ over HR through the down-regulation of important factors in the process such as *FOXM1*, *RAD51*, *RAD54L*, and *NEIL3* among others (*51*–*56*). In addition, *PLXND1* was down-regulated upon miR-99a-5p silencing in our setting. A study has reported that *PLXND1*(^−/−^) mice displayed defective germinal center reactions in T cell–dependent immune activation and defective production of IgG1 and IgG2b (*34*). Collectively, these conclusions were validated by miR-99a-5p involvement in promoting CSR in ex vivo splenic B cells. In our hands, silencing miR-99a-5p resulted in a significant decrease in CSR exhibited by lower IgG1 detection in antagomir-99a-5p–treated cells. In contrast, overexpressing miR-99a-5p resulted in a significant increase in CSR in mimicker-99a-5p–treated cells. EV-mediated RNA transfer continues to be a subject of debate, particularly regarding the efficiency of cargo delivery (*57*, *58*). While (i) recently showing that murine CSR is regulated by intercellular signaling via EV noncoding RNA among B cells (*59*) and (ii) our current findings suggesting a role for miR-99a-5p CSR regulation within the melanoma TME, we recognize that providing definitive evidence for EV-mediated delivery remains technically challenging. To resolve this issue more conclusively, future studies incorporating in situ RNA tracking and genetic manipulation of donor and recipient cells will provide additional support to this emerging intercellular signaling cascade.

Our miRNA pull-down assay identified two targets of miR-99a-5p in B cells, *HJURP* and *SOX5*, both of which have been independently shown to negatively affect CSR (*35*). *HJURP* plays a role in chromatin remodeling and is involved in the DNA damage response (DDR) pathway through promotion of HR repair pathway (*60*). *SOX5*, as a transcription factor, regulates gene expression of select genes and has been shown to regulate B cells proliferation and differentiation into plasmablasts, a short-lived lower-affinity antibody-producing cells (*61*). Hence, *HJURP* and *SOX5* are likely suppressing CSR through distinct but complementary mechanisms. These findings go hand in hand with the conclusions we drew from all the earlier data showcasing miR-99a-5p role in regulating the cell cycle and DDR pathways, resulting in enhanced CSR.

Our data suggest that B cell EVs–derived miR-99a-5p exerts its function by promoting a positive immunotherapy response possibly via two arms, (i) the regulation of the cell cycle and DNA repair pathways in B cells and, subsequently, (ii) through CSR promotion to induce a diverse antibody repertoire. Switched B cells were reported to be enriched in the tumors of immunotherapy responders with high clonal expansion (*62*, *63*). Increased BCR class switching and affinity maturation and BCR variable region diversification in responders were identified as an independent OS prognostic factor in melanoma (*62*, *63*), suggesting that B cells actively participate in shaping antitumor immunity. These observations were validated in our hands, where ICB therapy responders showed a significant increase in isotype class-switching in comparison to nonresponders. We were able to further link that observed phenotype to a miR-99a-5p–mediated mechanism. By analyzing Ig isotype frequency in our human B cells, we were able to show that (i) the data from both settings mirrored each other in the context of IgA switching being more significant than IgG and (ii) that miR-99a-5p could potentially regulate CSR, even at the transcriptomic level. The significance of these results becomes particularly pertinent when considering their potential on patient survival. These observations were additionally validated with our OS analysis based on *CD19*, *CD20*, *IgM*, *IgG*, and *IgA* expression in independent melanoma TCGA dataset. High expression of *CD19* and *CD20* is associated with significantly better OS. Nonetheless, what was notable is that Igs had a more declared positive outcome, with IgG (presenting a switched phenotype) exhibiting higher correlation than IgM with OS. The potential difference between our data shown in Fig. 5 (C and D) and the OS analysis in Fig. 5E regarding the correlation of Igs with outcomes may be explained by differences in dataset composition. The SKCM dataset is derived from a heterogeneous cohort of patients with melanoma, including individuals at various disease stages, undergoing different treatment regimens, analyzed at different time points and sampled from both primary and metastatic sites. In contrast, our cohort consists of pretreatment baseline samples exclusively from primary tumors, providing a more controlled but narrower context for comparison.

In addition, high expression of *HJURP* has been linked to poor OS in different cancers (*64*) . As we have shown, *HJURP* is a direct target of miR-99a-5p and its inhibition results in enhanced CSR (*35*). In melanoma, lower expression of HJURP is correlated with significantly better OS (Fig. 5D). These findings suggest that B cell–derived EVs core function is to regulate B cells themselves, at least in the case of miR-99a-5p, to promote B cell maturation, antibody diversification, and antitumor B cell population. We demonstrate, in a recent study, that under physiological conditions, B cells can regulate CSR via an EV-mediated intercellular transfer of functional noncoding RNA cargo via the mir5099-ROD1-AID axis (*59*). In addition, several other reports on B cell–derived EVs show that they can carry p-MHC complexes (*65*), functional BCRs (*66*), and functional IgM (*67*) and IgG (*68*), which in turn can be uptaken by recipient cells to modulate their functions. This strongly suggests a possible role for B cell–derived EVs in immunosurveillance through B cell maturation and antigen presentation. We propose that B cell EVs may act at several levels: (i) miR-99a-5p, identified in B cell EVs in responders, promotes CSR in situ and, enhances the antitumor immune response in responders. By expressing and packaging miR-99a-5p into their EVs, B cells manage to enhance their role in the immune response by promoting the diversification of their antibody repertoire. We hypothesize that B cells with a more mature profile could induce unswitched B cells to undergo CSR through transfer and uptake of EVs. This hypothesis remains to be explored because of limitations in studying EV transfer in situ. Nonetheless, we have shown in an independent setting the ability of murine B cells to promote CSR through intercellular EV transfer in vitro (*59*). We have also shown that a previously uncharacterized miRNA (miR-5099) in combination with a set of long ncRNAs are carried within B cell EVs and could contribute to antibody diversification (*59*). (ii) In addition, we propose that B cell EVs could play a role in T cell activation, exemplified by restored CD69 expression in CD8^+^ T cells in the absence of B cells. This was corroborated in an in vitro tumor-killing assay and the observed B cell EV uptake in T cells. B cell EV–mediated activation of CD8^+^ T cells can explain, at least in part, the role B cell EVs play in T cell cytotoxicity against cancer cells, possibly independently of EV-mi-R99a-5p. (iii). Last, another interesting observation is that B cell EVs did not induce cell death when incubated alone with melanoma cells, suggesting that B cell EVs induce melanoma cell death through intermediate cells (i.e., T cells), or by making the melanoma cells more susceptible to immune-targeting, which are interesting avenues for future exploration.

Together, this suggests that B cell–derived EVs may function (i) through inter–B cell communication and (ii) more broadly, by influencing other immune cell types within the TME, including T cells. These interactions could enhance antigen presentation, modulate effector functions, and create a more immunogenic environment conducive to tumor rejection. This supports the notion that, in addition to other immune cells within the TME, B cells might play a crucial, albeit underexplored, role in shaping immunotherapy outcomes. With this understanding, the potential of B cells should be considered when designing future anticancer therapies to fully leverage their role in antitumor activity. In addition, identifying a key regulator of B cell response in immunotherapy responders, such as miR-99a-5p and potentially *HJURP*, could transform our approach to immunotherapy—shifting the focus toward the entirety of the immune TME, rather than solely on established immune cells as the main drivers of the immune response.

## MATERIALS AND METHODS

### Patient samples and tissue processing

Melanoma slow-frozen patient biopsies were obtained from the biobank of the Dermatology Department of the University Hospital Zürich (KEK_PB_2018-00194). All samples used were surplus materials from routine surgeries (table S1). Collection, preparation, and freezing of the fresh biopsies are outlined in (*69*). Informed consent had been obtained from all patients and all experiments conducted according to the ethical rules of the Cantonal Ethic Committee of Zurich (Ethics form BASEC: 2014-0425). Tissue processing protocol is detailed in (*27*). Briefly, cryovials were thawed in 37°C water bath and tissue fragments were collected into a precooled 35-mm dish. Tissue fragments were washed and sliced into smaller pieces in 1 ml of digestion enzyme mixture of Dispase-II (5 mg/ml final concentration) and deoxyribonuclease I (10 μg/ml final concentration). Tissue fragments and digestion supernatant were collected into 2-ml Eppendorf tube and incubated at 37°C with shaking in a heat block for 2 hours. Samples were centrifuged at 500*g* for 5 min to clear EVs from cells and other debris. Supernatant containing the EVs was collected into a fresh tube and kept at 4°C until analysis.

### Tissue biopsy flow cytometry

Tissue fragments were further processed by incubating them with 1 ml of collagenase-IA (0.5 mg/ml final concentration) at 37°C with shaking in a heat block for 45 min. Samples were centrifuged at 500*g* for 5 min to separate cells from EVs. After centrifugation, cell suspension was passed through 70-μM prewetted cell strainer. With the back of 5-ml syringe plunger, the remaining tissue was mildly dissociated, and an additional 5 ml of media was used to wash the cells through. Single-cell suspension was then stained for fluorescence-activated cell sorting (FACS) analysis and stained with Live/Dead blue (Invitrogen, L23105) and antibodies against CD45 (BioLegend, 304012), CD19 (BioLegend, 302233), and CD3 (BioLegend, 300305). Samples were acquired using Cytek Aurora 5L.

### EVs isolation and characterization

Digestion supernatant was serially centrifuged to clear from cells and debris. Supernatant was centrifuged for 10 min at 300*g* and 2000*g*. Supernatant was then centrifuged at 10,000*g* for 30 min before ultracentrifugation at 100,000*g* for 1 hour at 4°C. After ultracentrifugation, EVs pellet was resuspended in 0.22 μm of filtered phosphate-buffered saline (PBS) for downstream analysis. EV characterization was carried out using TEM and NanoFCM. With TEM, we were able to visualize the morphology and size of EVs isolated from processed tissue samples. For TEM, EV samples were transferred onto pioloform-coated EM copper grids by floating the grids on a droplet containing freshly prepared EV suspension placed on parafilm and incubated for 5 min. The grids were washed three times for 5 min each before contrasting bound EVs with a mixture of 2% w/v methyl cellulose and 2% w/v uranyl acetate (9:1 ratio) on ice for 10 min. Grids were then allowed to air-dry before imaging. TEM images were analyzed with MAPS software (Thermo Fisher Scientific) for EV sizes estimation. The same samples were sized with nanoFCM sizing beads made of 68-, 91-, 113-, and 155-nm beads cocktail. Both size estimations were compared by grouping TEM-sized particles into the four size categories of nanoFCM beads (0- to 75-nm–sized particles are assigned to 68-nm category, 76 to 98 nm to 91-nm category, 99 to 130 nm to 113-nm category, and 131 to 180 nm to 155-nm category). EV processing and downstream characterization followed MiSEV guidelines (*70*).

### EVs surface marker analysis

We aimed at characterizing surface and cargo molecules of tissue-derived EVs from responding and nonresponding patients with melanoma undergoing ICB therapy. To characterize EVs surface proteins, we used a multiplex beads assay for the detection of 37 broad markers simultaneously, including several immune markers to provide an overview of the EVs landscape (MACSPlex EV Kit IO, Miltenyi Biotec). Briefly, 120 μl of resuspended EVs samples were incubated with 15 μl of EV capture beads overnight at room temperature in Thermomixer orbital shaker (Eppendorf). EVs and bead mixture was then washed with 500 μl of MACSPlex buffer and centrifuged at 3000*g* for 5 min at room temperature. EV-bead conjugates were then incubated with 15 μl of EV detection reagent (5 μl of CD9, CD63, and CD81 detection reagents) for 1 hour at room temperature in Thermomixer orbital shaker (Eppendorf). Samples were washed with 500 μl of MACSPlex buffer and centrifuged at 3000*g* for 5 min at room temperature. Five hundred microliters of the supernatant was discarded, and the rest of the samples were resuspended and transferred into FACS tubes for downstream analysis. Blank controls (beads and Macsplex buffer) were used to deduct background signal (fig. S2). Samples were analyzed using LSRII Fortessa 4L FACS machine.

### Immunostaining and Nanoflow analysis

For immunostaining EVs, isolated EVs samples were stained in 100 μl of antibody staining solution at a dilution of 1:100 for 1 hour on ice. Antibodies against melanoma (melanoma cocktail, Novus, NBP2-34547B), CD63 (BioLegend, 353005), and CD45 (BioLegend, 304012), were used. In addition, EVs were stained with CT (1:1000, C34564). Excess antibodies and CT were washed out to reduce background signal by topping up samples with 1 ml of 0.22 μm of filtered PBS before ultracentrifugation at 100,000*g* for 1 hour at 4°C. The supernatant was discarded, and the EV pellets were resuspended in 50 μl of 0.22 μm of filtered PBS for downstream analysis. Experimental controls included PBS blank controls, antibody staining solution without EVs, and EVs stained with isotype controls. These controls were prepared to exclude any background signal or nonspecific binding of antibodies (fig. S2). The samples were acquired using the nanoanalyzer, NanoFCM. Alignment was adjusted in every experimental setup using 250-nm QC nanosphere beads at a concentration of 2.07 × 1010 particles/ml (Lot 2012141, nanoFCM). Silica sizing beads (S16M-Exo, nanoFCM) composed of four bead populations of 68-, 91-, 113-, and 155-nm sizes were used to estimate the size of recorded particles. All beads were diluted at 1:100 for machine setup. Laser power was kept at 20 and 40 mW for the 488- and 640-nm lasers, respectively. Sampling pressure was kept at 0.8 kPa, and the samples recorded were kept at 2000 to 12,000 events/min. Concentration and size estimation of all samples were obtained using NF profession V2.18 software, and downstream analysis was carried out using Flowjo software.

### EVs subpopulation isolation and sequencing

To understand the role of melanoma and B cell–derived EVs in shaping the immune TME, in situ EVs isolated from processed tumor tissue were analyzed. Isolated EVs were sorted according to their cell origin using exosome-streptavidin isolation/detection reagent (Thermo Fisher Scientific, USA). Briefly, biotinylated primary antibodies against melanoma (antibody cocktail against MART-1, tyrosinase, and gp100 (Novus, NBP2-34547B) and CD19 (Invitrogen, 13-0199-80) were conjugated to streptavidin-coated Dynabeads magnetic beads (Invitrogen, 10608D) for 1 hour at room temperature. After this, preenriched EVs were incubated with the conjugated Dynabeads for 18 to 20 hours at 4°C. EVs conjugated to beads were removed from the magnet and lysed with TRIzol for 5 min at room temperature. The samples were put on the magnet again to collect the magnetic beads. The supernatant containing EVs cargo was collected, and total RNA was extracted with RNeasy Mini Kit (Qiagen, 74104). The concentration and purity of total RNA were assessed using NanoDrop2000. Quality control of RNA samples was performed with Agilent 2100 Bioanalyzer RNA 6000 Nano Kit. For library preparation and sequencing, miRNA-seq was prepared using the UMI small RNA library kit (BGI Genomics). Differential expression analysis was done using DESeq2. Cutoff for significant miRNA was set at Log_2_FC ≥ 1, and FDR < 0.05. miRNAs target prediction and biological process functional analysis was done with miRnet 2.0 (*71*).

### Tumor-killing assay

Patient-derived melanoma cell line M161022 was counted and stained with CellTrace Yellow (C34567, Invitrogen) for 20 min. Excess dye was quenched with complete culture media. Cells were pelleted and resuspended in fresh complete media and seeded in a 12-well plate at a density of 1 × 10^5^ cells per well. The plate was incubated overnight to allow cell attachment. At day 1, PBMCs isolated from healthy donors were thawed, counted, and either left whole (complete PBMC condition) or depleted from B cells using StemCell EasySep Human CD19 Positive Selection Sample Kit II (depleted PBMC condition). PBMCs were seeded in a 24-well plate at a density of 5 × 10^5^ to reach an effector:target (E:T) ratio of 5:1 and allowed to rest for 2 hours. PBMCs were then left unstimulated or stimulated with SEB (50 ng/ml) alone or with SEB and antagomiR/negative control (300 nM). After activation for 4 hours, PBMCs were transferred to the wells containing melanoma after aspirating the existing media. PBMCs and melanoma cells were cocultured for 48 hours, after which with samples were analyzed with FACS to estimate cell populations and melanoma cell death. Briefly, all cells were washed and collected by centrifugation at 500*g* for 10 min. Samples were and immunostained with an antibody cocktail of CD45 (BioLegend, 368525), CD19 (BioLegend, 302233), CD3 (BioLegend, 300425), and CD69 (BioLegend, 748763), for 1 hour on ice. Samples were washed with PBS and centrifuged at 500*g* for 10 min. Cell death was assessed with annexin V–fluorescein isothiocyanate (FITC, BioLegend, 640945) and 7-AAD (Invitrogen, 00-6993-50) staining for 15 min at room temperature before doing a final wash with PBS and centrifuging at 500*g* for 10 min. Samples were acquired using Cytek Aurora 5L. For EVs-rescue setup, PBMCs from the same donors were thawed for B cells isolation with StemCell EasySep Human CD19 Positive Selection Sample Kit II. B cells were stimulated with SEB (50 ng/ml) and incubated for 48 hours. B cells were pelleted, and their conditioned medium containing EVs was centrifuged for 10 min at 300*g* and 2000*g*. Supernatant was then centrifuged at 10,000*g* for 30 min before ultracentrifugation at 100,000*g* for 1 hour at 4°C. After ultracentrifugation, EV pellet was stored at 4°C for downstream assays.

### EVs uptake assay

For melanoma-EV uptake, the same setup as above was followed. For B cell–EV uptake, PBMCs were thawed and counted to obtain a dilution enough for a set number of wells, from which B cells were isolated using StemCell EasySep Human CD19 Positive Selection Sample Kit II. Remaining PBMCs were seeded in a separate plate and allowed to rest. B cells were then stained with CellTrace Yellow (C34567, Invitrogen) for 20 min. Excess dye was quenched with complete culture media. B cells were allowed to rest for 2 hours. B cells and the rest of PBMCs were stimulated with SEB (50 ng/ml). After activation for 4 hours, B cells were mixed with the rest of the PBMCs and transferred to plates preseeded with melanoma cells (E:T ratio of 5:1) after aspirating the existing media. PBMCs and melanoma cells were cocultured for 48 hours. Samples were analyzed with FACS to analyze CT uptake in non–B cells. Briefly, all cells were washed and collected by centrifugation at 500*g* for 10 min. Samples were and immunostained with an antibody cocktail of CD45 (BioLegend, 368525), CD19 (BioLegend, 302233), and CD3 (BioLegend, 300425) for 1 hour on ice. Samples were washed with PBS and centrifuged at 500*g* for 10 min. Cell death was assessed with annexin V–FITC (BioLegend, 640945) and 7-AAD (Invitrogen, 00-6993-50) staining for 15 min at room temperature before doing a final wash with PBS and centrifuging at 500*g* for 10 min. Samples were acquired using Cytek Aurora 5L. For nanoFCM validation of CT^+^ EVs secretion, melanoma, and B cells were CT-stained, independently. After 48 hours, conditioned medium was collected and EVs isolation was done using the previously mentioned method.

### miRNA silencing

For miRNA silencing, we used miRCURY LNA miRNA Power Inhibitors (Qiagen) as an antagomiR against miR-99a-5p (ACAAGATCGGATCTACGGGT). In 500 μl of complete culture media, targeted cells were incubated with antagomiR-99a-5p at a final concentration of 300 nM for 4 hours to allow for uptake through gymnosis without use of transfection reagents. An antagomiR control with no hits of >70% homology to any sequence in any organism in the National Center for Biotechnology Information (NCBI) and miRBase databases was used as negative control (TAACACGTCTATACGCCCA). The duration of the full experiment (72 hours) allows for efficient uptake and activity of power inhibitors.

### PBMCs bulk RNA-seq

PBMCs from healthy donors were thawed, washed twice with media, and resuspended in wash buffer [2% fetal bovine serum (FBS) 1 mM EDTA PBS]. Cell suspension was separated into two tubes for B and T cells isolation using StemCell EasySep Human CD19 Positive Selection Sample Kit II or EasySep Release Human CD3 Positive Selection Kit, respectively. Isolated B and T cells were seeded in 48-well plates and allowed to rest for 2 hours before stimulation with SEB (50 ng/ml) for 4 hours. Isolated cells were treated with antagomir-99a-5p or negative control at a final concentration of 300 nM for 48 hours. Cells we collected, washed, and lysed with TRIzol before total RNA isolation with RNeasy Mini Kit (Qiagen, Germany). The concentration and purity of total RNA were assessed using NanoDrop2000. Quality control of RNA samples was performed with Agilent 2100 Bioanalyzer RNA 6000 Nano Kit. Library preparation and sequencing was done with DNBSEQ platform (BGI Genomics). DEGs analysis was done using DESeq2 on Dr. Tom platform (Beijing Genomics Institute, BGI). Cutoff for significant genes was set at Log_2_FC ≥ 1, and *q* value < 0.05. Ridgeline analysis of enriched biological process was carried out for the significant up-regulated genes. X axis shows expression fold change of antagomir-99a-rp–treated cells over negative control antagomiR. Ridgeline analysis was done with ExpressAnalyst (*72*).

For IGH frequency estimation, the frequency of each Ig heavy chain gene isotype (IGHM, IGHA1, IGHA2, IGHG1, IGHG2, IGHG3, IGHG4, IGHD, and IGHE) was calculated as the ratio of normalized counts at each isotype to the total normalized counts at all Ig heavy chain genes in each sample.

### CSR assay

Splenic primary B cells from female C57BL/6 mice were cultured in RPMI 1640 media with 20% fetal calf serum (Sigma-Aldrich), 5% Medium NCTC-109 (Thermo Fisher Scientific), 1× penicillin-streptomycin-glutamine (Gibco), 1 mM sodium pyruvate (Gibco), 1× MEM-NEAA (Gibco), 2 mM GlutaMAX (Gibco), and 55 μM β-mercaptoethanol (Pan Biotech). To induce CSR from IgM to IgG, primary B cells were activated with lipopolysaccharide (25 μg/ml) and interleukin-4 (10 ng/ml). Cells were treated with 1 μM final concentration of miR-99a-5p silencer and mimicker, in addition to their respective negative controls. Cells were incubated for 96 hours. At termination points, cells were collected and washed two times with cold FACS buffer with centrifugation at 400*g* for 4 min at 4°C. Cells were stained with anti-Mo IgG (Invitrogen), anti-Mo IgM (Invitrogen), and Zombie Violet Fixable Viability (BioLegend) for 30 min, in the presence of Fc block (BD Biosciences). Cells were washed two additional times and centrifuged before resuspension in PBS. Samples were acquired using Cytek Aurora 5L.

### miRNA-mRNA pulldown

PBMCs from healthy donors were thawed, washed twice with media, and resuspended in wash buffer (2% FBS and 1 mM EDTA PBS). B cells were isolated using StemCell EasySep Human CD19 Positive Selection Sample Kit II. Isolated B cells were stimulated with SEB stimulation with SEB (50 ng/ml) and electroporated with a biotinylated mimicker of miR-99a-5p and biotinylated negative control. Cells were lysed with RLT lysis buffer (Qiagen) to release mRNA-miRNA/biotin complexes. Lysed cells supernatant was incubated with streptavidin-coated Dynabeads magnetic beads (Invitrogen) for 18 to 20 hours at 4°C. Samples were put on the magnet to collect the mRNA-miRNA/Biotin complexes conjugated to magnetic beads. Collected mRNA was stored at −80°C for subsequent sequencing. All data were normalized to their respective controls in the DEG analysis, and transcripts significantly higher in miR-99 mimic were considered for downstream analysis.

### Immunostaining and quantitative pathology

Immunophenotyping was performed using a six-color multispectral immunofluorescence protocol. Briefly, 3-μm-thick FFPE tissue sections were mounted on HistoBond adhesive microscope slides (Marienfeld, Germany). Slides were baked at 70°C for 5 hours, deparaffinized and rehydrated through serial ethanol concentrations. Next, they were placed in tris-EDTA (pH 9.0) buffer and heated at +95°C for 80 min for antigen retrieval and then blocked using 4% bovine serum albumin (BSA)/0.01% Triton X-100/tris-buffered saline (TBS). Antibodies against CD45 (Dako, M0701), IgG (Abcam, ab307524), IgM (Dako, A0425), and IgA (Southern Biotech, 2050-07) were used and diluted in 1% BSA/0.01% Triton X-100/TBS. After overnight incubation with primary antibodies at 4°C, the slides were rinsed, and the tissue sections were incubated with secondary antibodies labeled with fluorophores (Jackson ImmunoResearch) for 1 hour at room temperature. Last, the slides were incubated with 4′,6-diamidino-2-phenylindole (Thermo Fisher Scientific) for 5 min at room temperature and then mounted using VECTASHIELD PLUS Antifade mounting medium (Vector Labs). Whole slides were imaged using PhenoImager HT 2.0 (Akoya), a multispectral image scanner and multispectral images of 20× high power field were captured. The images were subjected to spectral unmixing techniques using Inform v. 2.7 (Akoya). The image data files were then converted into flow cytometry data format using Biobase and Flowcore (R studio), and further analysis to identify and quantify distinct cell populations was performed through Flowjo v.10.10.0 software.

### Processing of bulk RNA-seq melanoma datasets

Bulk RNA-seq melanoma datasets from Hossain *et al.* (*19*), Hugo *et al.* (*20*), and Riaz *et al.* (*21*) were downloaded from NCBI Gene Expression Omnibus (GEO) under the accession numbers GSE213145, GSE78220, and GSE91061, respectively. Raw sequencing fastq files from each of the three datasets were downloaded using the package sratools (v 3.0.3), and their primary quality control was performed using the software FastQC (v 0.11.9). Next, in each of the files, the package Cutadapt (v 1.18) was used to trim the adapters, filter low-quality bases from the 3′-end of the reads (average base quality threshold >15) and remove reads shorter than 35 base pairs and with more than 20 unidentified bases. Following this step, the secondary quality control of the files was carried out to validate Cutadapt results. The reads were then mapped to the human GENCODE GRCh38.p13 reference genome using the splice-aware aligner GSNAP (v 2021-08-25) ensued by the implementation of package RSeQC (v 5.0.1) for the file mapping quality check. Mapping quality control comprised the verification of mapped read distribution across different genome regions and the percentage quantification of ribosomal RNA contaminants. Packages MultiQC (v 1.14) and Samtools (v 1.10) were used in summarizing the file reports from each step and manipulating the sam/bam files, respectively. Last, the table of the raw read counts was generated through the function featureCounts (v 2.0.3) for each of the samples passing the quality controls. Differential gene expression analysis of melanoma datasets was performed using the DESeq2 (v 1.38.0) package in R (v 4.2.3). Batch correction of the read counts was implemented with the ComBat function in sva (v 3.48.0) package. Immune infiltration estimation was carried out using immune deconvolution method TIMER2.0 (*73*) based on bulk RNA-seq data. Protein-protein interaction network analysis was done using NetworkAnalyst (*74*). IMEx Interactome was selected as reference database.

### OS analysis

OS analysis of independent melanoma patients (SKCM dataset from TCGA) was carried out using GEPIA platform (*75*). OS was calculated on the basis of gene expression of denoted genes using the Mantel to Cox test for hypothesis test. Group cutoff is based on quartile (75 to 25%) to separate high/low expression groups, Confidence interval (95%) is shown as a dotted line on the survival plots.
