# Supplementary material for: B cell extracellular vesicles influence melanoma response to immune checkpoint therapy
Source: Sci Adv. 2025 Oct 8;11(41):eadt4551. doi: 10.1126/sciadv.adt4551 (PMC12506976; doi:10.1126/sciadv.adt4551)
Supplement: Supplementary file 1 — Figs. S1 to S4 Tables S1 to S3 [file sciadv.adt4551_sm.pdf]

Science Advances
AAAS

Supplementary Materials for

B cell extracellular vesicles influence melanoma response to immune checkpoint therapy

Ala'a Al Hrout *et al.*

Corresponding author: Richard Chahwan, richard.chahwan@gmail.com

Sci. Adv. **11**, eadt4551 (2025)
DOI: 10.1126/sciadv.adt4551

This PDF file includes:

Figs. S1 to S4
Tables S1 to S3

Supplementary Figures

Fig. S1

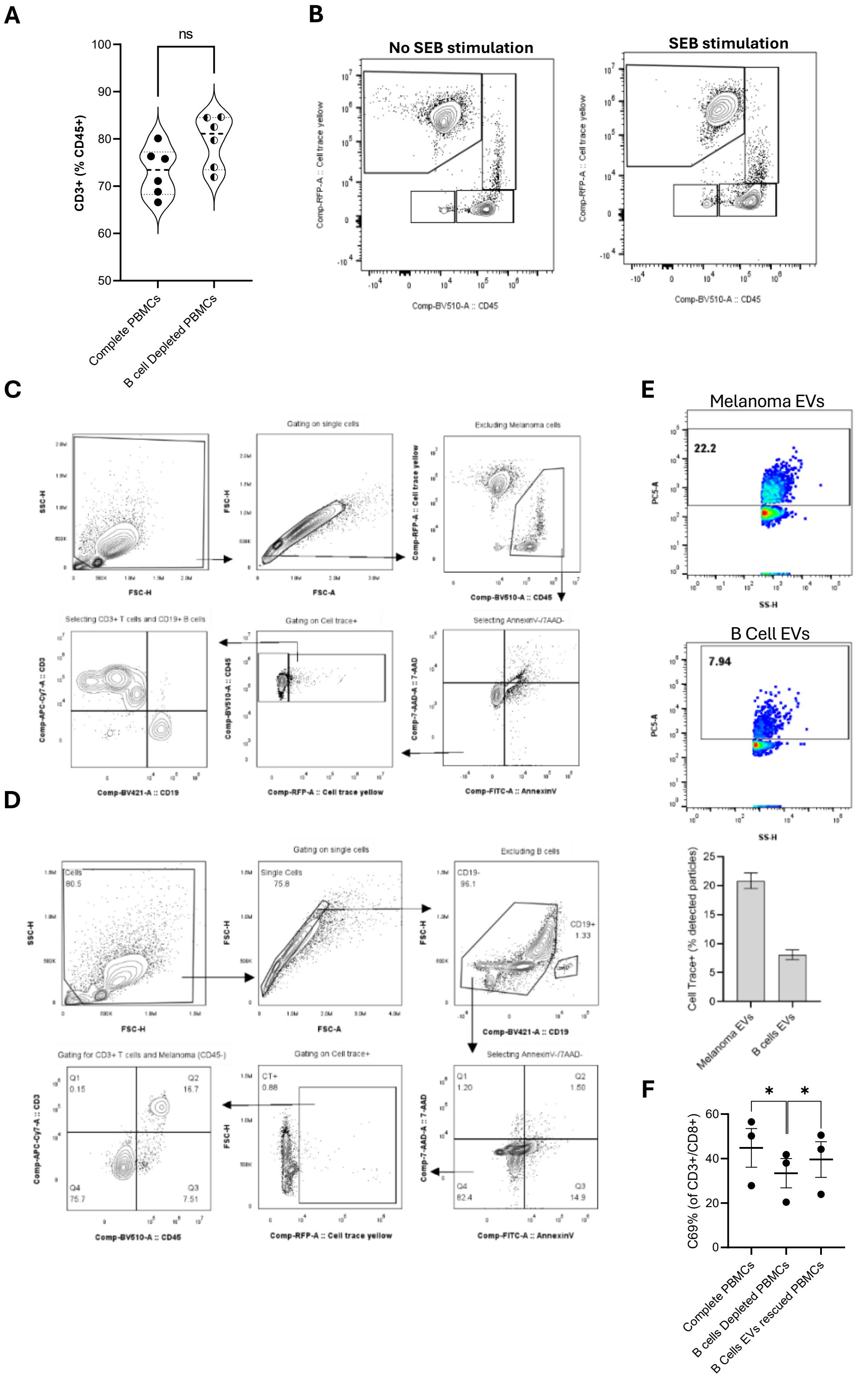

Figure S1. (A) $CD3^+$ T cells in complete and B cell-depleted cultures, with or without SEB stimulation. Presented as percentage of $CD45^+$ population. *P* values were determined by paired t-test (B) Representative FACS plots of cell trace-stained Melanoma co-culture and (C) gating strategy. (D) gating strategy for cell trace-stained B cells co-culture. (E) Representative FACS plot of EVs isolated from cell trace-stained Melanoma cells and cell-trace stained B cells, with their quantification. Samples were acquired with nano-analyzer NanoFCM. (F) $CD69^+$ cells in complete, B cell-depleted, B cell EVs-rescued PBMC cultures. Presented as percentage of $CD45^+/CD3^+/CD8^+$ population. *P* values were determined by paired t-test (*$p<0.05$).

Fig. S2

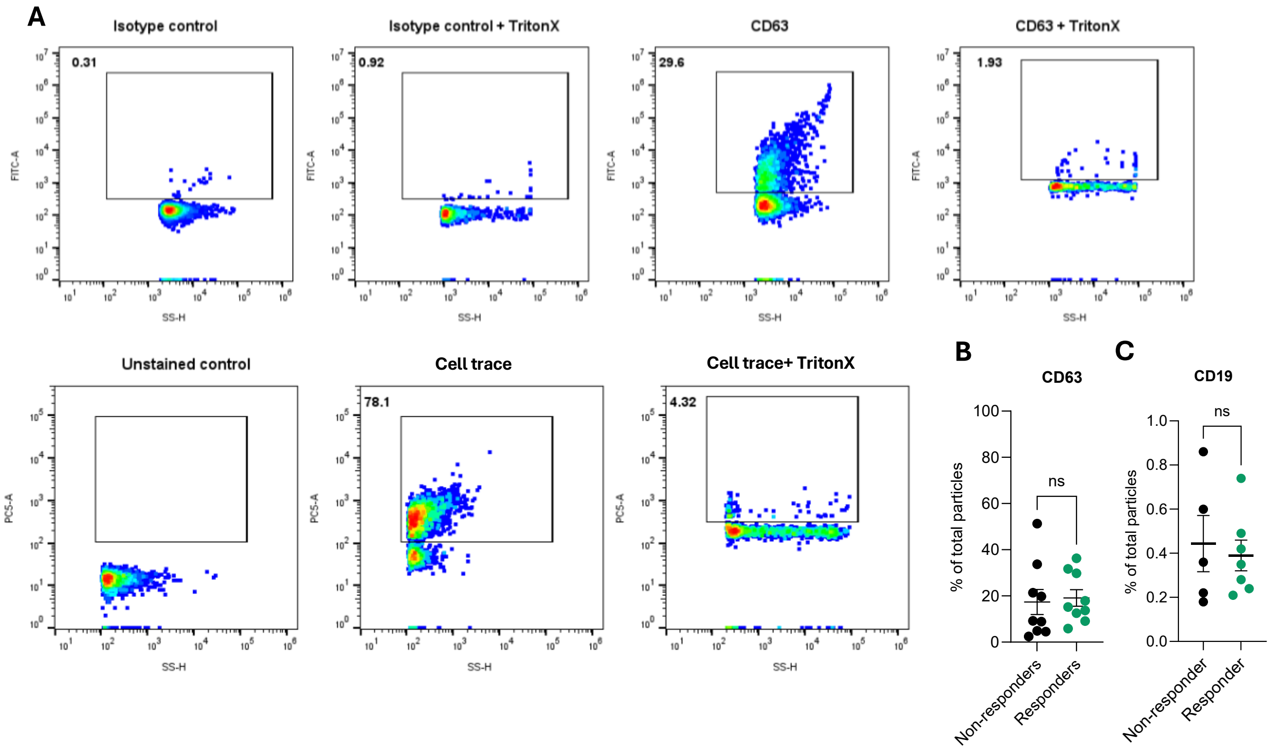

Figure S2. (A) Representative FACS plots for EVs detection controls. Unstained control, cell trace to stain EVs membranes, Isotype control, CD63 antibody staining and their respective triton-X controls. Samples were acquired with nano-analyzer NanoFCM. (B) $CD63^{+}$ EVs, presented as percentage of detected particles in responders (green, n=9) and non-responders (black, n=6). P values were determined by Welch's t-test. (C) $CD19^{+}$ EVs, presented as percentage of detected particles in responders (green, n=7) and non-responders (black, n=5). P values were determined by Welch's t-test.

Fig. S3

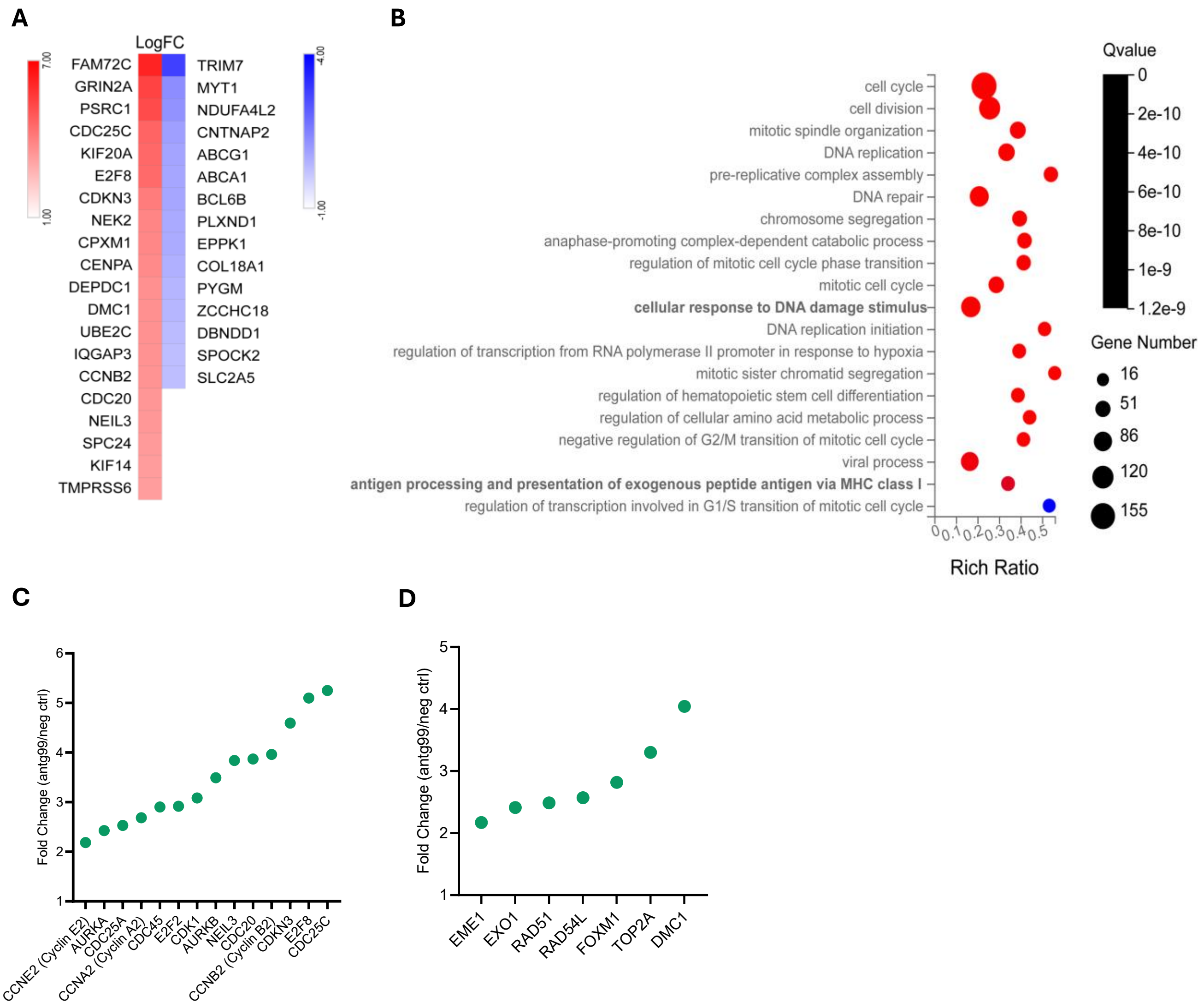

Figure S3. (A) Expression heatmap of the top 20 DEGs in antagomiR-99a-5p or negative control antagomiR. (B) bubble chart of enriched biological process terms of significant upregulated genes in antagomiR-99a-5p treated B cells in comparison to negative control antagomiR. Analysis and bubble chart graphs were done using ShinyGO 0.77 (80). (C) Expression of cell cycle genes in antagomiR-99a-5p treated B cells presented as fold change over negative control antagomiR, based on RNAseq. (D) Expression of DDR and HR genes in antagomiR-99a-5p treated B cells presented as fold change over negative control antagomiR, based on RNAseq.

Fig. S4

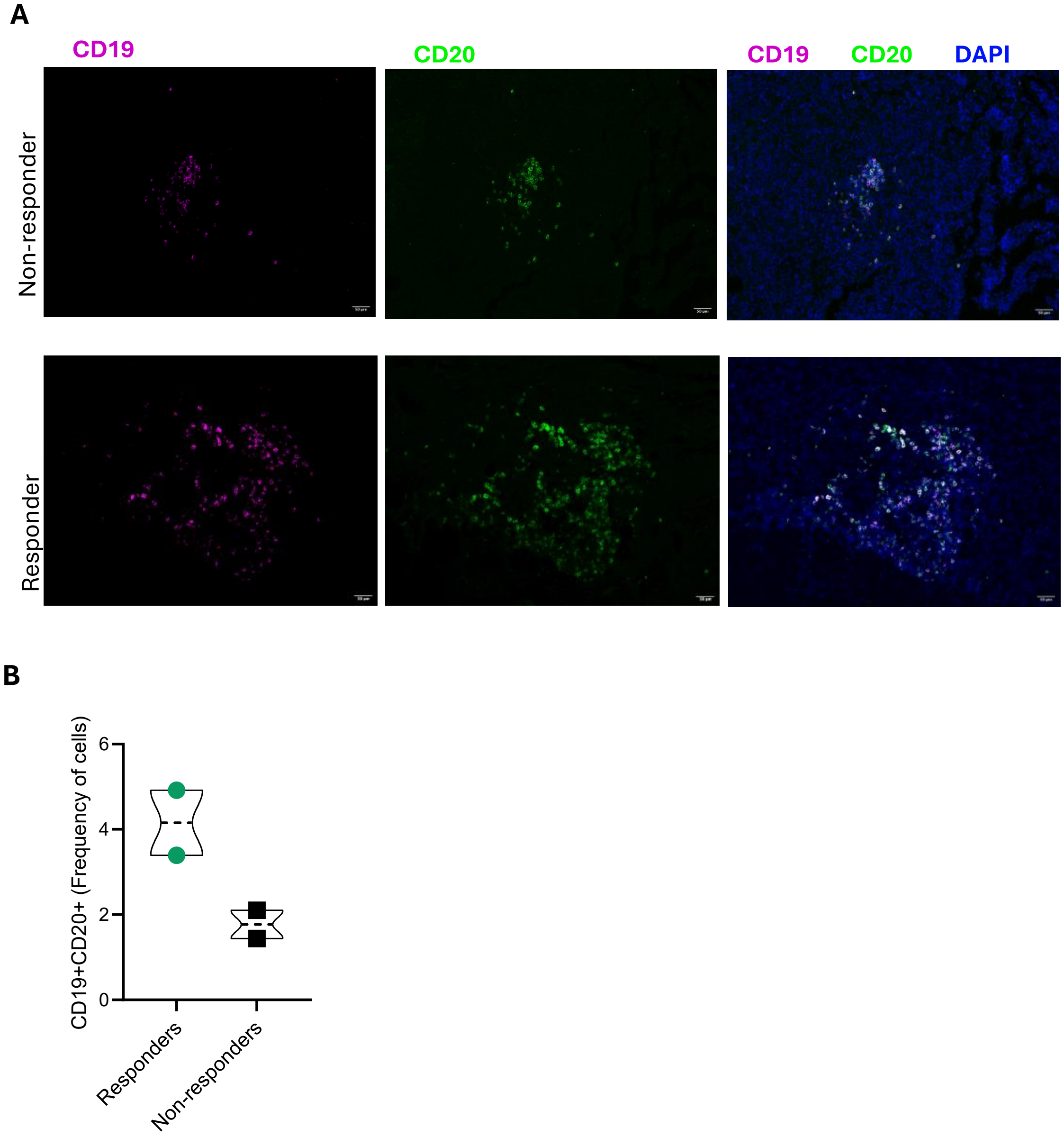

Fig. S4. (A) Representative images of immunofluorescence staining for CD19 (magenta) and CD20 (green), with DAPI (blue) from responders and non-responders (scale bar= 50 um). (B) quantification of CD19+CD20+ cells in responders and non-responders tissues, represented as frequency of cells (n=2).

Supplementary Table S1

	Response to anti-PD1 treatment	Overall Survival (days)	Anti-PD1 Treatment	sex	Age	Disease stage
P1	Responder	2677	Pembrolizumab	F	77	IV
P2	Responder	1285	Pembrolizumab	M	53	IV
P3	Responder	231	Nivolumab	M	66	IV
P4	Responder	2200	Ipilimumab+Nivolumab	M	56	IIIC
P5	Responder	2013	Ipilimumab+Nivolumab	M	65	IIIC
P6	Responder	1728	Nivolumab adj.	M	58	IIIC
P7	Responder	1548	Pembrolizumab	F	81	IV
P8	Responder	1111	Pembrolizumab	F	84	IIIC
P9	Responder	547	Nivolumab	M	65	IV
P10	Non-responder	1922	Pembrolizumab	M	70	IV
P11	Non-responder	2123	Pembrolizumab	F	64	IV
P12	Non-responder	246	Pembrolizumab	F	84	IV
P13	Non-responder	76	Nivolumab	M	78	IIIC
P14	Non-responder	687	Nivolumab adj.	M	77	IIID
P15	Non-responder	98	Ipilimumab+Nivolumab	M	59	IV
P16	Non-responder	9	Nivolumab	M	85	IV
P17	Non-responder	215	Nivolumab adj.	F	65	IV
P18	Non-responder	142	Pembrolizumab	M	86	IV

Supplementary Table S2

Pathway	Total	Expected	Hits	P.Value	FDR
Biological Process					
B cell activation	194	5.78	38	1.03E-20	6.05E-19
Adaptive immune response based on somatic recombination of immune receptors built from immunoglobulin superfamily domains	218	6.5	37	5.31E-18	1.61E-16
B cell differentiation	82	2.44	19	2.43E-12	3.76E-11
Humoral immune response	157	4.68	25	6.53E-12	8.92E-11
REACTOME					
Antigen Activates B Cell Receptor Leading to Generation of Second Messengers	32	1.39	18	5.21E-17	2.37E-14
Signaling by the B Cell Receptor (BCR)	199	8.67	36	1.20E-13	3.26E-11
Downstream Signaling Events Of B Cell Receptor (BCR)	173	7.54	23	1.43E-06	4.53E-05
KEGG					
B cell receptor signaling pathway	71	3.03	28	1.23E-20	3.92E-18

Supplementary Table S3

Gene Symbol	log2 (antg99_B / Neg_ctrl_B)	Qvalue (antg99_B / Neg_ctrl_B)
'PIF1'	1.179573978	0.04980
'PDSS2'	0.445197115	0.04964
'TTC17'	-0.270760546	0.04939
'DECR1'	0.4123713	0.04935
'DCAF12'	0.744942976	0.04932
'KIF27'	-0.550418696	0.04920
'TNFRSF13C'	-0.328040306	0.04912
'COX6A1'	0.430056014	0.04912
'R3HDM2'	-0.27932292	0.04912
'CSRNP1'	-0.527079553	0.04912
'DNAJC11'	0.305512433	0.04899
'TARS1'	-0.243007599	0.04884
'HSD17B10'	0.513546563	0.04880
'ATXN1L'	-0.285476625	0.04872
'PDP1'	-0.415837583	0.04872
'AEN'	0.417279819	0.04872
'ZNF740'	-0.244791959	0.04851
'GPRIN1'	0.61068529	0.04849
'RNF13'	-0.381990446	0.04819
'KDM6B'	-0.542518981	0.04809
'ST3GAL1'	-0.350077261	0.04809
'XPNPEP1'	0.358742528	0.04798
'RTRAF'	0.309157128	0.04796
'CLPTM1L'	0.256125187	0.04796
'HIVEP3'	0.494026982	0.04781
'C2orf68'	-0.281043381	0.04774
'HDAC1'	0.237340503	0.04774
'DNAJA3'	0.286292291	0.04752
'C11orf24'	0.248992237	0.04752
'LRRC3'	-0.615863691	0.04752
'CEBPB'	-0.639280881	0.04751
'UTP20'	0.375444852	0.04744
'MAGOH'	0.395495117	0.04740
'RICTOR'	-0.555070669	0.04725
'NDUFA9'	0.36283047	0.04725
'RB1CC1'	-0.492801907	0.04725
'FEM1B'	-0.252811003	0.04720
'ZFYVE16'	-0.475476929	0.04720
'MTA2'	0.191141831	0.04718
'KHNYN'	-0.270692078	0.04717
'CAPRIN2'	-0.504513123	0.04713

'PSMA6'	0.369666084	0.04699
'EI24'	0.381338007	0.04699
'SCAF4'	-0.203963184	0.04684
'ZNF84'	-0.368052684	0.04678
'KLF9'	-0.453471652	0.04674
'COX7B'	0.418713166	0.04655
'ST14'	-0.428467946	0.04631
'TMEM134'	-0.440812866	0.04631
'VARS1'	0.541019326	0.04601
'OLA1'	0.439032358	0.04594
'ZNF800'	-0.441640131	0.04569
'SRC'	0.529936795	0.04546
'CNPY3'	-0.202517109	0.04543
'MRTO4'	0.384776683	0.04543
'GLMN'	0.456842899	0.04543
'RCN1'	0.469711182	0.04543
'MAP3K2'	-0.361696717	0.04540
'THAP9'	-0.605391922	0.04540
'KARS1'	0.281891608	0.04526
'LETM1'	0.217019519	0.04518
'KIFC2'	-0.496073348	0.04516
'ALDOC'	0.524812702	0.04514
'KLHL9'	-0.317914299	0.04514
'CCND2'	0.820771368	0.04514
'CREB1'	-0.274454544	0.04513
'IDNK'	-0.471300585	0.04506
'SF3B5'	0.37701907	0.04506
'PTPN6'	0.477306739	0.04499
'ZMYM2'	-0.422900137	0.04499
'SETD1B'	-0.369574029	0.04492
'SUB1'	0.462575655	0.04483
'MCTP2'	-0.306283664	0.04478
'TRIO'	-0.347526343	0.04478
'DCUN1D2'	-0.469797507	0.04462
'PDZD11'	0.420799528	0.04460
'ZXDC'	-0.366978719	0.04458
'TRMT2A'	0.208386936	0.04450
'CHST14'	-0.442792438	0.04447
'SUN2'	-0.447855557	0.04447
'ABCA2'	-0.531521798	0.04439
'NDUFS7'	0.315901371	0.04439
'IRS1'	-0.633229275	0.04433
'GORASP1'	-0.369047514	0.04428
'RBBP8'	0.332172427	0.04412
'ST7L'	-0.643102257	0.04398

'RNASEH2A'	0.434930367	0.04395
'WDR3'	0.358446967	0.04395
'C17orf67'	-0.639111255	0.04395
'SARS2'	0.406266653	0.04395
'NR1H2'	-0.226998211	0.04395
'ZNF354B'	-0.430104586	0.04385
'SCARB1'	0.71237799	0.04385
'SDHD'	0.314547374	0.04382
'SLBP'	0.382333103	0.04372
'OGFRL1'	-0.499719079	0.04359
'RNF220'	-0.263954672	0.04358
'GPBP1'	-0.201277929	0.04358
'ZNF292'	-0.28075951	0.04358
'GPI'	0.421533844	0.04356
'MPV17L2'	0.459767319	0.04356
'ING4'	-0.352594383	0.04356
'MTHFD2'	0.415044901	0.04342
'SNX11'	0.462804514	0.04342
'YIF1B'	0.313070113	0.04342
'RFC4'	0.470828348	0.04321
'TP73'	0.962054387	0.04317
'TADA2B'	-0.230325657	0.04317
'NAE1'	0.337874612	0.04307
'HECA'	-0.315241675	0.04300
'VDR'	0.937414382	0.04290
'RIMKLB'	-0.367027093	0.04271
'NEDD9'	0.499311956	0.04264
'BUB3'	0.324704091	0.04264
'SETD6'	-0.337252751	0.04232
'TSPYL2'	-0.46438067	0.04188
'EBNA1BP2'	0.521486303	0.04186
'PSEN1'	-0.285795447	0.04186
'MT2A'	1.241377678	0.04186
'SLC25A15'	0.526713117	0.04171
'CSNK1G2'	-0.350455942	0.04171
'ATP5MJ'	0.512622943	0.04160
'GPC1'	-0.769558309	0.04155
'ANP32E'	0.280220345	0.04155
'GARRE1'	-0.389332067	0.04146
'CRTC1'	-0.534254136	0.04098
'EXOSC7'	0.390955437	0.04089
'KIF24'	1.010738178	0.04089
'NDUFB8'	0.376428446	0.04089
'ERCC6L'	1.018981436	0.04089
'N4BP2L2'	-0.327807866	0.04087

'CAP1'	0.333923017	0.04087
'TAGAP'	-0.290888246	0.04087
'WASHC4'	-0.346403522	0.04087
'DPH2'	0.319665488	0.04073
'OGFOD1'	0.345590572	0.04051
'STX7'	-0.307900166	0.04016
'CPOX'	0.35594183	0.03995
'CCDC186'	-0.448769559	0.03995
'TMEM220'	-0.851201689	0.03975
'LOC112694756'	0.723178665	0.03967
'FEM1C'	-0.317514838	0.03966
'DDX39A'	0.235950302	0.03941
'PSMD14'	0.41471463	0.03935
'MSH6'	0.291958914	0.03935
'PSMA5'	0.366636587	0.03935
'SAMD4B'	-0.248266406	0.03928
'RPA1'	0.328579733	0.03927
'PPP4R3A'	-0.302995755	0.03924
'CORO7'	-0.224909298	0.03897
'NUDT21'	0.210230912	0.03883
'SH3GLB2'	-0.21277532	0.03865
'COX8A'	0.459967055	0.03858
'SAYSD1'	-0.523422441	0.03855
'FKBP1A'	0.346478177	0.03851
'MCAT'	0.644190275	0.03844
'NDUFS4'	0.479129198	0.03834
'SMCR8'	-0.285432441	0.03834
'PSME2'	0.614780079	0.03834
'MVD'	0.307608221	0.03832
'TCP11L1'	-0.275280982	0.03830
'RFX3'	-0.547343587	0.03810
'MRPL13'	0.431006348	0.03781
'TMX4'	-0.472854168	0.03769
'TGIF1'	-0.403674633	0.03769
'NDUFA6'	0.523376231	0.03734
'EIF2B3'	0.588854499	0.03730
'DCLRE1A'	0.390137792	0.03730
'GPR82'	1.329679724	0.03725
'SKIL'	-0.3894617	0.03719
'KANSL1'	-0.345172332	0.03697
'EXOSC3'	0.392582279	0.03697
'CYCS'	0.55060184	0.03697
'SNX2'	-0.334732183	0.03694
'S1PR1'	-0.606955773	0.03684
'BCL6B'	-1.258487666	0.03683

'NAPA'	0.221597976	0.03683
'TRAPPC1'	0.41198797	0.03675
'CDK17'	-0.356240461	0.03668
'PSME3'	0.360745843	0.03662
'OGG1'	0.320650646	0.03662
'GNL2'	0.30228152	0.03650
'PUM2'	-0.272122947	0.03647
'DAZAP1'	0.211269553	0.03636
'SCP2'	0.351981866	0.03636
'ATOX1'	0.496947257	0.03629
'PRNP'	-0.321518031	0.03622
'MZF1'	-0.385982273	0.03622
'NUP37'	0.605565585	0.03622
'SIRT1'	-0.372969477	0.03615
'NRBP2'	-0.53958406	0.03613
'DCAF8'	-0.283586752	0.03612
'EDEM3'	-0.309889229	0.03610
'PGM2'	0.347164691	0.03586
'CLCN6'	-0.350080736	0.03582
'DENND2D'	-0.393940991	0.03576
'FRS2'	-0.385743177	0.03566
'ZNF83'	-0.362322741	0.03566
'PDLIM1'	0.39047476	0.03566
'TTF2'	0.47676246	0.03560
'RBM33'	-0.349707293	0.03560
'NDUFS3'	0.440322631	0.03554
'CLTC'	0.221467048	0.03551
'RSBN1'	-0.292925743	0.03546
'BCCIP'	0.330184442	0.03543
'KIF18A'	0.87128009	0.03540
'PREB'	0.260517209	0.03528
'CHCHD3'	0.487526768	0.03528
'SSR4'	0.313639071	0.03528
'GPR35'	-0.820690266	0.03510
'ZBTB11'	-0.256964562	0.03508
'GLG1'	-0.219630729	0.03508
'MDM4'	-0.636628185	0.03495
'ZRANB1'	-0.388715485	0.03492
'SIVA1'	0.338564948	0.03492
'ATP5MF'	0.432982545	0.03492
'HARS1'	0.217844178	0.03476
'AK9'	-0.545079132	0.03461
'CENPW'	0.972711213	0.03461
'CINP'	0.592854174	0.03461
'PSMB3'	0.395301723	0.03461

'USP5'	0.345047323	0.03461
'BOD1L1'	-0.377700993	0.03457
'VDAC3'	0.391305245	0.03453
'UROD'	0.397293491	0.03453
'CBR1'	0.392318354	0.03453
'LSM1'	0.373458406	0.03452
'UQCR10'	0.399895446	0.03452
'HSPA1A'	-0.622269584	0.03452
'ANP32B'	0.237069351	0.03450
'PDSS1'	0.666112037	0.03437
'WTAP'	-0.226325388	0.03430
'NF1'	-0.421515338	0.03427
'CCT3'	0.408089066	0.03427
'LARGE1'	-0.815660019	0.03426
'HSPE1'	0.521956638	0.03403
'ADGRB2'	1.821813909	0.03387
'CDKN2D'	-0.852939321	0.03372
'DLAT'	0.34046376	0.03372
'CPXM1'	2.10352101	0.03372
'CXCL16'	-0.755971947	0.03362
'RRP1B'	0.335141906	0.03360
'KCTD7'	-0.456112061	0.03352
'ZNF860'	-0.497808929	0.03352
'LIG1'	0.293122378	0.03352
'POLR3K'	0.484687141	0.03352
'TBL1X'	-0.37369503	0.03352
'VRK1'	0.355563065	0.03352
'DBNDD1'	-1.04242066	0.03352
'SGPL1'	-0.396179531	0.03352
'PIEZO1'	-0.27228522	0.03352
'UQCRC1'	0.313292455	0.03351
'GRIN2A'	2.622132565	0.03338
'CHMP1B'	-0.436954181	0.03338
'MRPS12'	0.485263073	0.03338
'BANF1'	0.366634318	0.03338
'NUP93'	0.428271256	0.03338
'TUBGCP5'	0.463216452	0.03331
'LGR4'	-0.809864828	0.03331
'EPPK1'	-1.20882538	0.03331
'MMS22L'	0.498243257	0.03329
'ZNF439'	-0.360673948	0.03317
'PMAIP1'	-0.329361053	0.03293
'HAGHL'	-0.692973283	0.03289
'FBXO28'	-0.286575996	0.03269
'MTMR3'	-0.275160131	0.03269

'YY1'	-0.261716625	0.03254
'ODC1'	0.479915845	0.03252
'ARPC2'	0.400664486	0.03240
'COA6'	0.433194171	0.03236
'MDH1'	0.391449925	0.03229
'MRPL28'	0.337249468	0.03209
'MEX3C'	-0.468882136	0.03209
'SMG1'	-0.243330213	0.03190
'MYT1'	-1.476518453	0.03190
'P2RY10'	-0.339295154	0.03158
'HNRNPA2B1'	0.19650522	0.03141
'NUDT15'	0.66457189	0.03140
'RPA3'	0.57937091	0.03140
'ATP5PD'	0.364359759	0.03138
'ABHD15'	-0.317895143	0.03123
'FXYD2'	1.253337863	0.03109
'RAB13'	0.822464695	0.03107
'OBSCN'	-0.506214148	0.03104
'TRIM38'	-0.322600028	0.03094
'ATP2B1'	-0.377021356	0.03094
'TCP1'	0.348139891	0.03094
'ZNF3'	-0.28916612	0.03092
'PSMC4'	0.246570103	0.03089
'CSRP1'	0.494633025	0.03081
'ZNF621'	-0.354836953	0.03049
'LMBR1L'	-0.572110764	0.03027
'PRKDC'	0.358217471	0.03027
'COPS3'	0.33466477	0.03027
'PSMD3'	0.24039411	0.03023
'TRAF1'	0.557502088	0.03023
'MRPL1'	0.584402576	0.03021
'DDB2'	0.419017932	0.03014
'DCAF13'	0.295999693	0.03014
'WSB2'	0.356203804	0.03014
'ZNF428'	0.440788023	0.03012
'RBM39'	-0.286367152	0.03012
'ITGAL'	0.495215602	0.02991
'ARL8A'	-0.384318565	0.02989
'HBP1'	-0.472573915	0.02983
'ATP5F1B'	0.434272855	0.02983
'CCDC174'	-0.34980937	0.02983
'NDUFA4L2'	-1.435647693	0.02983
'KLF11'	-0.528526727	0.02983
'ARHGAP45'	-0.404803325	0.02974
'DEPDC1'	2.016442227	0.02974

'DPM1'	0.312744886	0.02974
'N4BP3'	-0.381570498	0.02955
'GNB5'	-0.334336726	0.02931
'ZNF518B'	-0.339069151	0.02931
'SP100'	-0.284225806	0.02927
'HNRNPA1'	0.450283303	0.02914
'YLPM1'	-0.247430838	0.02914
'ELOC'	0.426253511	0.02914
'PDE4B'	-0.261513193	0.02914
'BACH1'	-0.509071049	0.02914
'ECT2'	0.839047362	0.02914
'WDR11'	-0.254902487	0.02914
'LANCL2'	0.467614056	0.02914
'TBC1D20'	-0.285420057	0.02894
'COQ2'	0.638045978	0.02894
'PSMC3IP'	0.698196448	0.02894
'SLC39A7'	0.247029659	0.02894
'EMSY'	-0.359193321	0.02894
'IDS'	-0.490158208	0.02851
'PCF11'	-0.337114577	0.02851
'CDK2AP2'	0.278006304	0.02836
'CSE1L'	0.431456186	0.02836
'CD55'	-0.499876112	0.02836
'DDX1'	0.37745621	0.02836
'CDCA4'	0.475226867	0.02836
'RAN'	0.491046384	0.02829
'AP1S1'	0.43967405	0.02803
'GNA11'	-0.404696786	0.02803
'ZNF471'	-0.433438539	0.02803
'ZNF33A'	-0.242805079	0.02803
'NDUFS5'	0.439238364	0.02803
'DDR1'	-0.596160258	0.02801
'ACTR3'	0.412629571	0.02792
'MAGOHB'	0.430609138	0.02792
'SEC61G'	0.389440943	0.02782
'PARP1'	0.310550227	0.02775
'CYP20A1'	-0.37591634	0.02768
'COX6C'	0.551599746	0.02760
'HIVEP2'	-0.429230147	0.02760
'YPEL5'	-0.404027581	0.02760
'PCMTD1'	-0.476689466	0.02758
'SRSF8'	-0.307891697	0.02741
'ZNF181'	-0.368673489	0.02735
'MRPS7'	0.44327007	0.02735
'S100A4'	0.826118473	0.02735

'ACSS1'	-0.458111216	0.02735
'TTBK2'	-0.387093618	0.02732
'BRI3BP'	0.332683198	0.02700
'YPEL2'	-0.731217005	0.02700
'PHKA2'	-0.232520424	0.02700
'NKD1'	-0.882559244	0.02700
'ZNF862'	-0.343600698	0.02689
'ZBTB4'	-0.316997897	0.02680
'CDKN1B'	-0.446994195	0.02678
'GPATCH8'	-0.258280841	0.02678
'SND1'	0.412782583	0.02678
'VMAC'	-0.510160607	0.02678
'ZNF655'	-0.264602747	0.02678
'AGPS'	0.323487983	0.02678
'BLNK'	0.201421352	0.02674
'PRMT3'	0.416131826	0.02671
'SGO1'	0.693204078	0.02671
'FAM199X'	-0.388913756	0.02670
'TKT'	0.450267127	0.02670
'DENND3'	-0.622974978	0.02667
'KIAA0895L'	0.519572972	0.02667
'TXN'	0.596074592	0.02667
'EEF1E1'	0.503216101	0.02667
'WDR1'	0.331479895	0.02667
'C16orf54'	-0.695323412	0.02664
'STMN1'	0.587137824	0.02664
'TRAPPC10'	-0.231550455	0.02663
'SLC38A5'	0.592159383	0.02661
'POU2F1'	-0.390303371	0.02655
'TPI1'	0.438097424	0.02655
'NCL'	0.355697653	0.02650
'POP7'	0.498046718	0.02646
'TTI2'	0.434855968	0.02646
'MLH1'	0.447994177	0.02640
'NDUFS2'	0.292548522	0.02640
'LYPLA1'	0.314332796	0.02638
'EP300'	-0.235888398	0.02638
'PTPA'	0.351344974	0.02638
'SECISBP2'	-0.310928718	0.02638
'CCDC136'	-0.689259672	0.02629
'RBX1'	0.374876581	0.02619
'HADH'	0.483867461	0.02611
'CCNL2'	-0.409872751	0.02611
'ABI2'	-0.332859293	0.02584
'ACAA2'	0.321192888	0.02562

'PRKCE'	-0.560471254	0.02562
'CARNS1'	-0.624420129	0.02562
'SAV1'	-0.292160675	0.02562
'TMEM156'	-0.514852007	0.02562
'TIMM21'	0.379557684	0.02562
'REXO5'	1.320817798	0.02551
'PLEKHM1'	-0.359319734	0.02549
'PHETA1'	-0.561981745	0.02541
'AFF4'	-0.438697199	0.02541
'EFTUD2'	0.303396431	0.02541
'NT5E'	-0.656221903	0.02523
'MARF1'	-0.430079956	0.02523
'OIP5'	1.027955689	0.02519
'ABCG1'	-1.279926265	0.02519
'PACS1'	-0.430196987	0.02512
'ISOC2'	0.555008848	0.02505
'CHAC2'	0.740009176	0.02499
'PSMB7'	0.399977047	0.02486
'BRWD1'	-0.46888719	0.02479
'SNRNP48'	-0.296448244	0.02470
'H6PD'	-0.368770936	0.02462
'ZC3H11A'	-0.281780268	0.02459
'BRD1'	-0.45534788	0.02450
'ATP5F1C'	0.395722813	0.02450
'PHB'	0.477535416	0.02450
'COMMD8'	0.408106913	0.02450
'EDEM1'	-0.466805488	0.02450
'ZCWPW1'	-0.509025363	0.02444
'CLK1'	-0.538924215	0.02441
'SLC25A5'	0.562940432	0.02441
'SLC35B1'	0.346627269	0.02422
'KLHL24'	-0.729863405	0.02422
'SYTL3'	0.757152259	0.02422
'ENTPD4'	-0.384416005	0.02422
'ACTB'	0.568322415	0.02416
'LINGO3'	-0.538483266	0.02411
'PRORP'	0.433032614	0.02411
'TIMM8B'	0.634151375	0.02407
'ABCB1'	-0.814997424	0.02358
'NEIL3'	1.941652808	0.02358
'KMT2E'	-0.334574096	0.02358
'CCT7'	0.364302266	0.02347
'PDHB'	0.328457357	0.02345
'CEBPG'	-0.402487516	0.02341
'CDC23'	0.391416978	0.02341

'FANCD2'	0.392861591	0.02332
'INTS9'	0.417102482	0.02332
'PPP1R15B'	-0.335892762	0.02332
'CBFA2T2'	-0.379357329	0.02332
'CHAF1A'	0.565600497	0.02330
'DUSP4'	0.534186542	0.02325
'ISYNA1'	-0.706980174	0.02321
'AKT3'	-0.461399351	0.02320
'SNRPA'	0.405239681	0.02316
'UBE2T'	0.940035036	0.02315
'TP53INP1'	-0.574117463	0.02315
'PRDX1'	0.394403083	0.02307
'FCRL2'	-0.390138258	0.02304
'PLEKHG1'	-0.29475753	0.02301
'IRAK2'	-0.502834426	0.02301
'HNRNPC'	0.308869786	0.02295
'RASGRP2'	-0.664966231	0.02272
'SAP30L'	-0.50911291	0.02266
'DIAPH1'	0.239604212	0.02252
'NPM1'	0.5163329	0.02209
'DBF4'	0.414956227	0.02208
'MAPK8'	-0.414149297	0.02205
'GINS4'	0.71205173	0.02205
'FIBP'	0.438846681	0.02205
'SIK3'	-0.394200061	0.02199
'UBQLN2'	-0.268914944	0.02199
'PSMD8'	0.219016589	0.02199
'NECTIN1'	0.827453158	0.02190
'MRPL20'	0.476622964	0.02174
'LSM3'	0.456393445	0.02151
'KAT6B'	-0.238601252	0.02150
'CDC14B'	-0.716442436	0.02150
'NOC2L'	0.264704629	0.02137
'LEO1'	0.324796321	0.02127
'PRDM1'	0.648400743	0.02122
'KMT2D'	-0.421527704	0.02121
'VAMP8'	0.333501397	0.02118
'MLLT6'	-0.198730472	0.02102
'SEPTIN11'	0.350729369	0.02089
'RIPOR2'	-0.529977992	0.02081
'HHEX'	-0.384182394	0.02072
'SLC2A5'	-1.010371374	0.02072
'CELSR1'	-0.605341267	0.02071
'MIPEP'	0.617618672	0.02061
'SMCHD1'	-0.443932359	0.02042

'PA2G4'	0.397575152	0.02035
'MYLIP'	-0.841591979	0.02029
'KIAA0232'	-0.436155144	0.02024
'ANXA6'	0.419933128	0.02019
'PGK1'	0.405529433	0.02019
'TBRG1'	-0.267361475	0.02019
'SEC11C'	0.510767232	0.02019
'SQLE'	0.386018957	0.02019
'HAUS1'	0.439246297	0.02014
'CORO2B'	-0.909656893	0.02009
'NATD1'	-0.513604461	0.02009
'SNRNP40'	0.429567612	0.02009
'POLD1'	0.348408246	0.02009
'ZDHHC12'	0.481667684	0.01999
'SNX16'	-0.567284978	0.01999
'COPA'	0.200657282	0.01993
'MRPL27'	0.48630394	0.01993
'POU2AF1'	-0.410288886	0.01988
'CCDC93'	-0.286166162	0.01968
'PSMB8'	0.549376851	0.01920
'EIF1'	-0.308014504	0.01919
'AHCY'	0.50737282	0.01919
'NANS'	0.424666841	0.01919
'NR2C2'	-0.398130991	0.01919
'DDB1'	0.245319124	0.01902
'ECHS1'	0.318177393	0.01902
'PRELID1'	0.481826497	0.01902
'HNRNPAB'	0.549237397	0.01902
'ATP5PO'	0.35774404	0.01890
'CCDC191'	-0.376013552	0.01863
'MEGF6'	-0.910440606	0.01833
'TNRC6B'	-0.402694563	0.01819
'LARS2'	0.389462794	0.01819
'ATXN3'	-0.436178991	0.01819
'PELI2'	-0.687128467	0.01817
'ETFA'	0.487784271	0.01813
'XRCC6'	0.315434418	0.01813
'RAC2'	0.631255787	0.01813
'EIF5A'	0.479231805	0.01797
'ATP2A2'	0.276550526	0.01797
'VPS25'	0.419660024	0.01797
'STOML2'	0.516159315	0.01782
'ACTG1'	0.639079261	0.01766
'HCN3'	-0.600179412	0.01753
'NDUFAB1'	0.541875999	0.01740

'PPM1G'	0.364289247	0.01740
'CLIC1'	0.517023	0.01733
'MYBL2'	1.550206439	0.01732
'SNN'	-0.827451608	0.01722
'KLF2'	-0.839756807	0.01716
'EIF4A1'	0.562487889	0.01716
'NEB'	-0.744382357	0.01716
'UNG'	0.546770475	0.01716
'SUCLG2'	0.527298179	0.01702
'RNF44'	-0.50673267	0.01670
'SMC2'	0.41601781	0.01653
'BRD4'	-0.364935948	0.01653
'CLN6'	0.423502461	0.01648
'SMAD5'	-0.357177357	0.01630
'STK38'	-0.48535448	0.01628
'IRF2BPL'	-0.454138802	0.01620
'ARPC1B'	0.373993944	0.01606
'COX5B'	0.391933375	0.01606
'PRPF4B'	-0.350210895	0.01606
'ADSL'	0.449055781	0.01603
'DMC1'	2.015009248	0.01600
'PAICS'	0.625182649	0.01587
'BAZ2A'	-0.395373575	0.01587
'CKS1B'	0.576609337	0.01587
'GET3'	0.358622783	0.01587
'NOP16'	0.497052588	0.01587
'ATP5PB'	0.433523595	0.01587
'FCMR'	-0.784655943	0.01584
'PDAP1'	0.294629793	0.01583
'YPEL1'	-0.6476236	0.01583
'UQCRFS1'	0.475867179	0.01582
'PSMD1'	0.31847361	0.01580
'SNTB1'	-0.733654417	0.01564
'MTFR2'	0.994377883	0.01562
'CTSF'	-0.810635094	0.01562
'PSMA3'	0.428610371	0.01559
'DSCC1'	1.399591953	0.01552
'CYC1'	0.310715088	0.01544
'OSTC'	0.421418784	0.01543
'YIPF4'	-0.279482846	0.01538
'CSNK1E'	-0.419640595	0.01533
'PFN1'	0.469912419	0.01497
'P4HB'	0.299605936	0.01489
'MKI67'	1.695527844	0.01486
'CHPT1'	-0.363173596	0.01479

'AK1'	-0.556789336	0.01462
'NNT'	0.351561355	0.01460
'SUMO3'	0.314964493	0.01451
'FURIN'	-0.32575142	0.01450
'TMPRSS6'	1.887098394	0.01447
'POLR2I'	0.5631191	0.01447
'MSL2'	-0.342060058	0.01447
'STIL'	0.849404028	0.01447
'SSRP1'	0.345689662	0.01447
'PSRC1'	2.569551795	0.01447
'TIMM17A'	0.517649549	0.01444
'HERC3'	-0.405946	0.01443
'BOLA2B'	1.622591506	0.01441
'C20orf27'	0.462901292	0.01429
'FAM43A'	-0.625841539	0.01414
'METTL7A'	-0.490996347	0.01414
'POLR2L'	0.44339715	0.01414
'RANBP1'	0.534532969	0.01414
'HSPD1'	0.609650182	0.01411
'MRPS11'	0.431938083	0.01411
'PIGS'	0.337043881	0.01411
'FAM72A'	0.87491374	0.01407
'CCDC86'	0.468020036	0.01407
'SRM'	0.53764419	0.01401
'RAB37'	-0.789557275	0.01397
'GTF3C6'	0.399633656	0.01385
'FDPS'	0.505157502	0.01385
'MAT2B'	-0.302727223	0.01385
'SLCO4A1'	0.37763771	0.01385
'PDIA3'	0.270688735	0.01385
'IL10'	1.163886656	0.01385
'HSPA14'	0.471267997	0.01385
'MAVS'	-0.262184834	0.01385
'MRPS15'	0.491461245	0.01385
'TGFBR2'	-0.348827244	0.01385
'TMEM259'	-0.31628943	0.01375
'RALGDS'	-0.383911884	0.01372
'CHD7'	-0.309407102	0.01371
'TRAIP'	0.726004059	0.01366
'NUP205'	0.20753714	0.01365
'DCLRE1B'	0.448989135	0.01365
'BRD3'	-0.413113848	0.01360
'FAM102A'	-0.426304502	0.01356
'DRG1'	0.353820681	0.01356
'PSMB1'	0.30818811	0.01356

'DAD1'	0.321461548	0.01347
'DEDD2'	-0.441104435	0.01332
'PIK3CA'	-0.42113609	0.01328
'ABCA1'	-1.264621892	0.01325
'ZDHHC2'	-0.340900725	0.01320
'SIK2'	-0.321538603	0.01320
'ANXA5'	0.30934612	0.01305
'ANLN'	1.590771164	0.01295
'ZNF274'	-0.406572074	0.01289
'CXCR4'	-0.742126944	0.01289
'RYK'	-0.387568664	0.01281
'GCLC'	-0.437128527	0.01252
'ELF1'	-0.31349645	0.01240
'ZNHIT1'	0.458799549	0.01230
'GSE1'	-0.661203626	0.01223
'MPDU1'	0.390898857	0.01222
'EIF4A3'	0.287205447	0.01216
'SLC16A1'	0.565013497	0.01208
'CKS2'	0.935604757	0.01197
'DYRK1A'	-0.301907979	0.01197
'OTUD1'	-0.645542006	0.01197
'POLDIP2'	0.438160702	0.01197
'GOT1'	0.379183153	0.01197
'IPO5'	0.438972525	0.01197
'AAGAB'	0.493815385	0.01197
'CRIPT'	-0.433078492	0.01197
'EPC2'	-0.363863345	0.01173
'LIMK1'	0.434582049	0.01159
'ZBTB7A'	-0.312871143	0.01159
'ATP5MC3'	0.577047013	0.01159
'TSPYL1'	-0.268606361	0.01159
'ANKRD44'	-0.395521623	0.01159
'RAPGEF1'	-0.203325925	0.01135
'TLE3'	-0.344166472	0.01135
'TRIP13'	1.069980085	0.01124
'CNOT9'	0.308564284	0.01121
'MYO19'	0.41117403	0.01119
'HIPK3'	-0.372968282	0.01115
'ICMT'	0.394154079	0.01115
'CCT5'	0.363009152	0.01112
'CHD2'	-0.356704067	0.01091
'SOX5'	1.327946598	0.01081
'ZNF585B'	-0.404347995	0.01075
'SLC25A53'	-0.611053311	0.01073
'KNL1'	0.870167528	0.01062

'CCR10'	1.137108037	0.01056
'CCNE2'	1.127969827	0.01056
'ARID5A'	-0.310538758	0.01055
'KLF12'	-0.452584195	0.01055
'ABTB2'	1.10793347	0.01055
'ZNF337'	-0.514225611	0.01055
'TEDC1'	0.546057671	0.01055
'RAB5IF'	0.437424724	0.01055
'SLC39A8'	0.564746992	0.01036
'DAPK2'	-0.796839844	0.01035
'LDHB'	0.682093319	0.01035
'NDUFS6'	0.560928977	0.01035
'LRATD2'	-0.862159479	0.01029
'NUB1'	-0.253759993	0.01029
'TNRC6C'	-0.486120394	0.01029
'CTDSP2'	-0.358432913	0.01027
'ERAL1'	0.32313316	0.01027
'MECP2'	-0.352522145	0.01027
'ARPC5L'	0.423906178	0.01027
'EMC8'	0.417224723	0.01021
'FAM117B'	-0.494410939	0.01021
'PYGM'	-1.110924329	0.01014
'ZNF431'	-0.315927354	0.01010
'PLEKHA1'	-0.70080947	0.01004
'SDHA'	0.260578193	0.01003
'SEM1'	0.506422372	0.01003
'CHURC1-FNTB'	0.812919852	0.00999
'SCCPDH'	0.478015208	0.00994
'CHD6'	-0.302423073	0.00991
'ATF7IP'	-0.365655183	0.00991
'MBP'	-0.365541824	0.00990
'SKA1'	1.333381646	0.00965
'PSMB6'	0.365693165	0.00965
'CAPZB'	0.297494324	0.00961
'CTPS1'	0.61413026	0.00960
'TRIB2'	-0.657971953	0.00960
'UTP4'	0.493866768	0.00951
'ALDOA'	0.341512551	0.00946
'CHST15'	-0.763668426	0.00944
'TPRG1L'	-0.39255531	0.00943
'VEZF1'	-0.405441969	0.00943
'PSMC5'	0.30000606	0.00938
'CS'	0.341138542	0.00936
'DUSP1'	-0.627946446	0.00936
'PSD3'	-0.718349498	0.00936

'TFDP1'	0.663216347	0.00936
'CARS1'	-0.342158069	0.00936
'EXO1'	1.269762897	0.00936
'RTCB'	0.415767001	0.00927
'CENPH'	0.575626412	0.00925
'PIK3CG'	0.40600974	0.00924
'ARL10'	-0.485183038	0.00921
'COX5A'	0.603275907	0.00921
'SOCS3'	1.122224625	0.00918
'CENPI'	0.965854868	0.00906
'DEPDC1B'	1.604845769	0.00906
'GBP1'	0.823928613	0.00904
'S1PR4'	-0.459615967	0.00904
'KLF3'	-0.841916996	0.00894
'SLC12A6'	-0.493163646	0.00890
'MRPL11'	0.521725252	0.00874
'IDH3A'	0.47362084	0.00863
'TSNARE1'	-0.436005519	0.00863
'SPOCK2'	-1.016464363	0.00851
'RUBCNL'	-0.517642757	0.00841
'POLA1'	0.683196887	0.00829
'ATAD2'	0.720627649	0.00819
'SGO2'	0.545962631	0.00819
'CEP120'	-0.325477177	0.00819
'NPM3'	0.804261816	0.00812
'ITSN2'	-0.305448352	0.00812
'ABRACL'	0.583461078	0.00809
'FAM214A'	-0.561316703	0.00798
'COL18A1'	-1.159566421	0.00793
'PRDM4'	-0.350220499	0.00785
'IL10RA'	0.371283772	0.00785
'NDUFV1'	0.480771169	0.00785
'PNPLA2'	-0.675034449	0.00785
'CKAP5'	0.365021998	0.00785
'IRF2BP1'	-0.375567651	0.00777
'TMEM97'	0.632973889	0.00765
'PIAS1'	-0.305529115	0.00756
'ZNF852'	-0.517190222	0.00755
'VIM'	0.583209972	0.00755
'CFL1'	0.487322409	0.00748
'NABP2'	0.569735203	0.00739
'RAP1GAP2'	-0.797319031	0.00738
'PPIF'	0.494239138	0.00733
'PRRC2B'	-0.302551744	0.00722
'DUT'	0.499372791	0.00714

'NME1'	0.835810294	0.00713
'SNRPE'	0.608609283	0.00710
'PKM'	0.596252879	0.00709
'KIF21B'	-0.576261651	0.00702
'HADHA'	0.346776342	0.00700
'DHFR'	0.802014073	0.00695
'SORBS3'	-0.559971962	0.00692
'GRAP'	-0.573921169	0.00692
'GMNN'	0.847858724	0.00689
'PGAM1'	0.562240295	0.00689
'ENO1'	0.558887454	0.00676
'ZNF532'	-0.300500693	0.00676
'ANAPC15'	0.581674584	0.00676
'MRPL12'	0.622559377	0.00676
'PNRC1'	-0.42463532	0.00673
'LTA'	0.775455138	0.00673
'LRRC59'	0.428009174	0.00673
'BACH2'	-0.622994195	0.00673
'AUNIP'	1.725533603	0.00673
'KIF15'	1.141189998	0.00672
'ABCB4'	-0.593588723	0.00669
'TAPT1'	-0.816789091	0.00663
'MGAT3'	0.5427467	0.00619
'FAM83D'	1.521233453	0.00618
'ARID5B'	-0.42520013	0.00615
'TXNIP'	-0.548397126	0.00609
'RAD51AP1'	0.915075613	0.00606
'NEIL1'	-0.801351306	0.00606
'PDIA6'	0.541223297	0.00606
'SEC11A'	0.269168755	0.00606
'FARSA'	0.407788247	0.00600
'MZB1'	0.687418355	0.00593
'LZTS2'	-0.491517755	0.00593
'CBX7'	-0.684249197	0.00578
'SMARCC2'	-0.296598579	0.00578
'CLTA'	0.311348326	0.00578
'KIAA2026'	-0.431494411	0.00578
'PTEN'	0.328788958	0.00578
'ESCO2'	1.320420727	0.00571
'JUN'	-0.855071354	0.00570
'ZNF783'	-0.390374048	0.00565
'RBM3'	0.307610033	0.00560
'RSU1'	0.35288582	0.00560
'RHBDF2'	-0.37032704	0.00549
'LRR1'	0.601184752	0.00543

'PSMC3'	0.465563959	0.00534
'MICOS10'	0.515571914	0.00532
'BZW2'	0.572380508	0.00528
'EFCAB14'	-0.23588855	0.00519
'DONSON'	0.673987798	0.00517
'ALAS1'	0.44933882	0.00508
'HIF1A'	-0.423883236	0.00497
'ATP11C'	-0.327340892	0.00496
'PARP8'	-0.402151882	0.00496
'RFTN1'	0.431470613	0.00488
'NUTF2'	0.4576523	0.00472
'SLC6A16'	-0.850172367	0.00467
'CLIC5'	0.951016112	0.00467
'CENPO'	0.563906729	0.00458
'BOLA3'	0.974861913	0.00453
'DNAJC9'	0.480535149	0.00453
'TCF20'	-0.258477226	0.00453
'FRAT1'	-0.656766524	0.00446
'URM1'	0.321831077	0.00446
'HELLS'	0.725068141	0.00439
'POLD3'	0.50999829	0.00436
'MTIF3'	-0.41203999	0.00435
'PNISR'	-0.414675163	0.00435
'MTCH2'	0.44494789	0.00434
'ARHGDIA'	0.241693731	0.00426
'FAM72B'	1.12465828	0.00418
'C1QTNF6'	-0.610100552	0.00416
'FUCA1'	-0.544207282	0.00414
'RASSF1'	-0.516669826	0.00414
'ASCC1'	0.609946137	0.00412
'MAPK1'	-0.285365324	0.00409
'TCP11L2'	-0.810154998	0.00409
'CNTNAP2'	-1.323610836	0.00409
'CCNL1'	-0.327102717	0.00409
'MAP3K21'	-0.715450937	0.00409
'MACROH2A1'	0.300321756	0.00409
'EIF2AK3'	-0.643050063	0.00396
'PMVK'	0.629357497	0.00394
'PBK'	1.591589382	0.00394
'KDM7A'	-0.906643581	0.00394
'TAF7'	-0.342304801	0.00391
'CYB5B'	0.499347732	0.00391
'TEDC2'	1.091808247	0.00389
'ASPM'	1.870329079	0.00389
'CREBBP'	-0.396511362	0.00388

'SNRPA1'	0.480561874	0.00388
'COX6B1'	0.522890814	0.00385
'HSPA5'	0.509202189	0.00385
'TLE5'	-0.534796971	0.00383
'NCAPD3'	0.576911018	0.00383
'ARPC4'	0.447276558	0.00382
'RAB29'	0.429123192	0.00382
'SLC30A4'	-0.651192026	0.00379
'CDK13'	-0.444237898	0.00376
'E2F2'	1.544833416	0.00375
'HMGB2'	0.633736998	0.00375
'MIDEAS'	-0.332545082	0.00375
'NOTCH1'	-0.555139373	0.00374
'CENPU'	1.091197347	0.00373
'WDHD1'	0.780605056	0.00372
'GLIPR1'	-0.701439491	0.00367
'SELL'	-0.623043253	0.00367
'TIMM13'	0.507441875	0.00358
'POU6F1'	-0.650450784	0.00357
'RFC3'	0.715314238	0.00355
'MEF2B'	0.795448258	0.00340
'MYADM'	-0.641433157	0.00336
'CDK4'	0.589033465	0.00335
'DPEP2'	-0.960718487	0.00328
'TBC1D9'	-0.56920631	0.00326
'FAM98A'	0.45948858	0.00326
'MRPL37'	0.565259173	0.00326
'DHRS13'	0.522689677	0.00323
'ORC6'	1.074531865	0.00323
'YWHAE'	0.521285996	0.00304
'BCL9L'	-0.624997648	0.00303
'FANCG'	0.564174342	0.00301
'PLD4'	1.001368566	0.00297
'ATAD3A'	0.459113034	0.00289
'YPEL3'	-0.740543042	0.00289
'TXN2'	0.551047207	0.00288
'TBXAS1'	-0.793037876	0.00288
'VMP1'	0.38990884	0.00279
'ASH1L'	-0.317535788	0.00279
'AARSD1'	0.536223479	0.00271
'MORC3'	-0.497176988	0.00269
'THOP1'	0.397105454	0.00269
'TPP1'	-0.248142239	0.00266
'PSMB5'	0.588765328	0.00266
'SLC1A4'	0.544001858	0.00266

'CCDC167'	0.650215357	0.00253
'FCGRT'	-0.57761818	0.00251
'RNF122'	-0.901440648	0.00250
'SIN3B'	-0.377661787	0.00250
'PTRHD1'	0.706804415	0.00249
'ZBTB18'	-0.61174172	0.00248
'FAM107B'	-0.319745802	0.00248
'FH'	0.563941822	0.00245
'TMOD2'	-0.327180594	0.00238
'EIF3C'	0.336400724	0.00238
'ELL2'	0.437381417	0.00233
'ZNF33B'	-0.420945296	0.00231
'ZEB1'	-0.358366761	0.00228
'TICRR'	1.283625809	0.00228
'MSH2'	0.461511502	0.00227
'SNRPD1'	0.543100176	0.00227
'RCC1'	0.576256058	0.00224
'XRCC2'	0.89958103	0.00222
'TAB2'	-0.302087295	0.00217
'ITGA1'	1.210039171	0.00217
'SCN3A'	-0.823119495	0.00217
'E2F1'	1.062502618	0.00207
'CHEK1'	1.010202533	0.00205
'PHF1'	-0.618240269	0.00203
'COQ3'	0.825353903	0.00201
'ZFP36L2'	-0.638617894	0.00201
'SPC25'	1.78487657	0.00201
'FBXO32'	-0.841980159	0.00195
'RACGAP1'	0.75985912	0.00195
'KMT2A'	-0.315250095	0.00188
'LSM4'	0.494620613	0.00188
'DTL'	1.327504267	0.00183
'SLC16A5'	-0.566956162	0.00183
'POC1A'	0.990875044	0.00177
'APEH'	0.502503314	0.00177
'SF3B1'	-0.241195799	0.00174
'GMPPB'	0.38561878	0.00172
'DIRAS1'	-0.59569033	0.00170
'PHF5A'	0.427248628	0.00168
'PFKP'	0.428714015	0.00164
'MND1'	1.531936879	0.00163
'PSMA2'	0.418537249	0.00162
'MXI1'	-0.955326548	0.00157
'MRPL51'	0.490645795	0.00153
'KNSTRN'	0.67467966	0.00153

'USPL1'	-0.44893885	0.00148
'CDT1'	1.257365081	0.00148
'GAPDH'	0.64080906	0.00147
'KIF22'	0.393554812	0.00146
'XRCC5'	0.396376248	0.00146
'DIAPH3'	1.432787843	0.00146
'CDK1'	1.625548297	0.00142
'SKA3'	1.587247573	0.00138
'ASF1B'	1.342381941	0.00138
'PRKCB'	-0.526108661	0.00138
'ITM2C'	0.870889593	0.00137
'CENPA'	2.077027186	0.00134
'APH1B'	-0.436516468	0.00134
'SKI'	-0.482687571	0.00131
'LMO2'	1.08373021	0.00126
'PSMB2'	0.476243571	0.00126
'CDC123'	0.473845331	0.00126
'HAT1'	0.543547221	0.00126
'TCF19'	0.90092295	0.00124
'NEK2'	2.116578393	0.00119
'FAM117A'	-0.527822878	0.00119
'CKAP2L'	1.717395584	0.00116
'ORC1'	0.973197385	0.00116
'JMJD1C'	-0.507134557	0.00115
'CEP126'	-0.938163537	0.00114
'ACOT7'	0.799392982	0.00114
'CD274'	0.982907883	0.00113
'POU2F2'	0.397733069	0.00112
'ZWILCH'	0.617848316	0.00112
'CNNM3'	-0.371625768	0.00111
'PBXIP1'	-0.618739233	0.00111
'CDCA7'	1.340667794	0.00111
'SAPCD2'	1.191604322	0.00106
'EME1'	1.119038895	0.00104
'RNF41'	-0.333021189	0.00099
'DLGAP4'	-0.308832575	0.00099
'STEAP1B'	1.523293071	0.00099
'CREBRF'	-0.635978506	0.00098
'PARPBP'	1.180985463	0.00098
'SAE1'	0.431388202	0.00098
'CIT'	0.909099202	0.00090
'RAD51'	1.315014687	0.00090
'CALM3'	0.446768862	0.00088
'CEP55'	1.646023193	0.00088
'CDCA2'	1.759975476	0.00087

'NCAPH'	1.39291582	0.00085
'POLR2G'	0.493684466	0.00085
'MARCHF1'	-0.737673887	0.00077
'GLRX'	0.647973548	0.00071
'CCNF'	1.061538189	0.00071
'KIF4A'	1.516444958	0.00070
'ACAT2'	0.540197293	0.00070
'MCM5'	0.325273176	0.00070
'NCAPG2'	0.73601507	0.00070
'ZCCHC18'	-1.099735943	0.00070
'SDF2L1'	0.739860358	0.00068
'PREX1'	-0.420381965	0.00068
'CENPE'	1.426628516	0.00067
'CALR'	0.520586393	0.00066
'TIMM23'	0.468531708	0.00065
'MACC1'	1.113474809	0.00065
'EVI2B'	-0.508137588	0.00061
'EZR'	-0.274594909	0.00061
'TRIM7'	-2.001386904	0.00060
'ZC3H6'	-0.646943009	0.00060
'BRCA1'	0.958199933	0.00060
'GTSE1'	1.632747064	0.00060
'TUBB'	0.796307122	0.00059
'FOCAD'	0.609716265	0.00059
'CALHM6'	-0.762073678	0.00059
'CENPM'	1.347476405	0.00059
'KLF7'	-0.737231035	0.00059
'ZNF107'	-0.50362374	0.00059
'MTURN'	-0.750099303	0.00057
'FERMT3'	0.364802156	0.00057
'H2AZ1'	0.587626711	0.00057
'GINS3'	0.719832824	0.00057
'CHCHD2'	0.442280061	0.00056
'PRR11'	1.459884984	0.00056
'BIRC5'	1.64156815	0.00055
'FANCA'	0.651811326	0.00055
'CENPX'	0.525272116	0.00054
'HASPIN'	0.82803872	0.00054
'KAT6A'	-0.404156133	0.00053
'TPM4'	0.595220064	0.00052
'WDR76'	0.842921721	0.00051
'FAM72C'	2.8148608	0.00050
'IQGAP3'	1.995242536	0.00050
'SPC24'	1.910615743	0.00050
'RFC2'	0.469538388	0.00046

'MYO1G'	0.439779262	0.00045
'AIFM1'	0.570730754	0.00045
'RAD54B'	0.960475432	0.00045
'NR1D2'	-0.695702418	0.00044
'CDC25C'	2.392884338	0.00042
'SNRNP25'	0.544728344	0.00037
'CDC25A'	1.339404771	0.00037
'NDC80'	1.426173714	0.00031
'KCNN3'	1.588333866	0.00031
'DNAJB11'	0.459413453	0.00029
'ADM2'	-0.787712212	0.00029
'CLSPN'	1.296243716	0.00029
'MTHFD1'	0.756409563	0.00027
'SEMA4A'	0.789519027	0.00027
'UQCRH'	0.566998394	0.00027
'AURKA'	1.27975356	0.00026
'ITPR1'	-0.46812714	0.00025
'DTYMK'	0.89042712	0.00025
'FOXM1'	1.494688662	0.00025
'KIF2C'	1.764844522	0.00025
'TSPAN14'	-0.67201826	0.00025
'SESN3'	-0.535681594	0.00024
'TUBA1C'	0.770272019	0.00024
'FOXRED1'	0.45592592	0.00024
'TYMS'	1.618780036	0.00024
'AURKB'	1.803974331	0.00024
'CDHR1'	1.631975081	0.00023
'PCLAF'	1.340845154	0.00022
'CCNB2'	1.987015947	0.00021
'HJURP'	1.691064366	0.00021
'BATF'	1.201235445	0.00020
'TSC22D3'	-0.759859127	0.00020
'IRAG2'	0.545419816	0.00020
'TMEM140'	-0.865992966	0.00020
'KLHL5'	-0.472973488	0.00020
'MCM3'	0.538127355	0.00019
'HNF1B'	1.264661643	0.00015
'NUF2'	1.470182316	0.00014
'PGGHG'	-0.570315392	0.00013
'PKMYT1'	1.495950841	0.00013
'MARCHF8'	-0.310899547	0.00013
'PRC1'	1.091146224	0.00013
'PPIB'	0.513968277	0.00013
'POLE2'	1.351425485	0.00012
'DYRK2'	-0.578551482	0.00012

'PCNA'	0.862003127	0.00012
'TUBA1B'	0.888934349	0.00012
'TROAP'	1.585643178	0.00011
'CDCA8'	1.308762937	0.00011
'E2F8'	2.350085212	0.00011
'POLQ'	1.350481691	0.00010
'POLA2'	0.685436901	0.00009
'NASP'	0.338549444	0.00008
'TMEM106C'	0.796135142	0.00008
'RECQL4'	1.055067114	0.00008
'CDKN3'	2.199765018	0.00008
'ZNF92'	-0.621063834	0.00008
'LCN10'	-0.885829842	0.00006
'CCNA2'	1.425395604	0.00006
'KIF14'	1.892530144	0.00006
'ZNF367'	0.922126213	0.00006
'BCAR3'	0.883891245	0.00006
'UBE2S'	0.740064089	0.00006
'MELK'	1.652406366	0.00005
'PPP5C'	0.452228162	0.00005
'PLK4'	1.157351647	0.00005
'INPP5D'	-0.248034842	0.00005
'BLMH'	0.421089267	0.00005
'SEMA4B'	-0.645680539	0.00005
'EPB41'	-0.520509783	0.00005
'HMGA1'	0.623983391	0.00005
'TTK'	1.5066565	0.00005
'CCNE1'	1.216650573	0.00004
'GNG7'	-0.673116196	0.00004
'LMNB2'	0.507268969	0.00004
'NUSAP1'	1.331159339	0.00003
'GINS1'	1.017685048	0.00003
'H2AX'	0.707180295	0.00003
'CCNB1'	1.389757692	0.00003
'PECAM1'	-0.655750099	0.00003
'EBP'	0.69540591	0.00002
'FANCI'	0.994953184	0.00002
'SHCBP1'	1.451909522	0.00002
'KIF18B'	1.653161496	0.00002
'MELTF'	1.207557598	0.00002
'RRM1'	0.883624095	0.00002
'PDIA4'	0.406194247	0.00002
'CREM'	0.676119747	0.00002
'KIF11'	1.420959064	0.00002
'KIF23'	1.741039957	0.00002

'MGAT5'	-0.407753195	0.00002
'NCF2'	0.799273635	0.00002
'C12orf75'	1.252859396	0.00002
'WDR62'	0.988542065	0.00002
'HMMR'	1.471226007	0.00001
'AMFR'	-0.362025755	0.00001
'IL21R'	0.774763446	0.00001
'DNMT1'	0.501259011	0.00001
'LGALS3'	1.160982032	0.00001
'MCM7'	0.520158386	0.00001
'MCM4'	1.035269378	0.00001
'GINS2'	1.363357226	0.00001
'KPNA2'	0.959352506	0.00001
'CHAF1B'	1.110242776	0.00001
'PAQR4'	1.080491471	0.00001
'FCRL4'	0.847141089	0.00001
'TTC21A'	-0.940573607	0.00001
'CDCA3'	1.502286266	0.00001
'CENPF'	1.345020181	0.00001
'RRM2'	1.724169285	0.00001
'PLXND1'	-1.223908657	0.00001
'TUBA4A'	0.508288606	0.00001
'SLC43A3'	0.91170923	0.00000
'GRAMD1C'	-0.771706696	0.00000
'ALYREF'	0.529173425	0.00000
'MCM6'	0.90230179	0.00000
'SPAG5'	1.196713676	0.00000
'TUBB4B'	0.567270421	0.00000
'LMNB1'	0.82822829	0.00000
'TUBG1'	0.857032846	0.00000
'PSMD2'	0.332046031	0.00000
'UBE2C'	2.00727147	0.00000
'LDHA'	0.82362595	0.00000
'ARHGAP11A'	1.302337836	0.00000
'PLK1'	1.801650239	0.00000
'CNR1'	-0.565467288	0.00000
'TPX2'	1.697796787	0.00000
'SKA2'	0.883176419	0.00000
'CDC6'	1.389668669	0.00000
'CDCA5'	1.602090841	0.00000
'MAD2L1'	1.102044723	0.00000
'MANF'	0.651653237	0.00000
'BUB1'	1.628230289	0.00000
'IDH2'	0.521869194	0.00000
'DLGAP5'	1.861976035	0.00000

'NCAPG'	1.683553642	0.00000
'UHRF1'	1.321419046	0.00000
'ZWINT'	1.499476357	0.00000
'NCAPD2'	0.641135236	0.00000
'ESPL1'	1.318376531	0.00000
'KIF20A'	2.366795333	0.00000
'FEN1'	0.971642255	0.00000
'IRF8'	-0.445174276	0.00000
'HCK'	0.789095408	0.00000
'IL6'	1.216386697	0.00000
'MCM2'	0.72218475	0.00000
'TOP2A'	1.722927399	0.00000
'RAD54L'	1.36294643	0.00000
'TK1'	1.61959813	0.00000
'BUB1B'	1.554475728	0.00000
'CDC45'	1.538350722	0.00000
'KIFC1'	1.504825971	0.00000
'GRAPL'	-0.858998737	0.00000
'MCM10'	1.537150361	0.00000
'PTTG1'	1.549187351	0.00000
'CDC20'	1.953002339	0.00000
'EZH2'	0.923082887	0.00000
'MACROD2'	1.740153661	0.00000
'PHF19'	0.917843117	0.00000
